# Granuloma-like structures in tuberculosis a critical review of their biology, quantitative behavior, and translational use

**DOI:** 10.1093/femsre/fuag053

**Published:** 2026-10-21

**Authors:** Enrico Mastrostefano, Giovanni Delogu, Alessandro Stamilla, Greta Segafreddo, Ivana Palucci, Giulia Degiacomi, Francesca Boldrin, Alessandro Ravoni, Davide Moretti, Elia Onofri, Michael Dal Molin, Jan Rybniker, Davide Sorze, Enrica Campagnaro, Laura Cioetto-Mazzabò, Paolo Tieri, Santiago Ramón-García, Maria Rosalia Pasca, Riccardo Manganelli

**Affiliations:** Istituto per le Applicazioni del Calcolo “Mauro Picone”, IAC – Consiglio Nazionale delle Ricerche, 00185 Rome, Italy; Department of Medicine and Surgery, University of Parma, 43121 Parma, Italy; Department of Biology and Biotechnology “Lazzaro Spallanzani”, University of Pavia, 27100 Pavia, Italy; Department of Molecular Medicine, University of Padua, 35121 Padua, Italy; Department of Basic Biotechnological Sciences, Intensivological and Perioperative Clinics, Università Cattolica del Sacro Cuore, 00168 Rome, Italy; Department of Laboratory and Hematological Sciences, Fondazione Policlinico Universitario A. Gemelli IRCCS, 00168 Rome, Italy; Department of Biology and Biotechnology “Lazzaro Spallanzani”, University of Pavia, 27100 Pavia, Italy; Department of Molecular Medicine, University of Padua, 35121 Padua, Italy; Istituto per le Applicazioni del Calcolo “Mauro Picone”, IAC – Consiglio Nazionale delle Ricerche, 00185 Rome, Italy; Istituto per le Applicazioni del Calcolo “Mauro Picone”, IAC – Consiglio Nazionale delle Ricerche, 00185 Rome, Italy; Istituto per le Applicazioni del Calcolo “Mauro Picone”, IAC – Consiglio Nazionale delle Ricerche, 00185 Rome, Italy; Department I of Internal Medicine, University of Cologne, 50937 Cologne, Germany; Department I of Internal Medicine, University of Cologne, 50937 Cologne, Germany; Department of Molecular Medicine, University of Padua, 35121 Padua, Italy; Department of Molecular Medicine, University of Padua, 35121 Padua, Italy; Department of Molecular Medicine, University of Padua, 35121 Padua, Italy; Istituto per le Applicazioni del Calcolo “Mauro Picone”, IAC – Consiglio Nazionale delle Ricerche, 00185 Rome, Italy; Department of Microbiology, Pediatrics, Radiology and Public Health, Faculty of Medicine, University of Zaragoza, 50009 Zaragoza, Spain; Research & Development Agency of Aragón Foundation (Fundación ARAID), 50018 Zaragoza, Spain; Spanish Network for Research on Respiratory Diseases (CIBERES), Carlos III Health Institute, 28029 Madrid, Spain; Department of Biology and Biotechnology “Lazzaro Spallanzani”, University of Pavia, 27100 Pavia, Italy; Fondazione IRCCS Policlinico San Matteo, 27100 Pavia, Italy; Department of Molecular Medicine, University of Padua, 35121 Padua, Italy

**Keywords:** tuberculosis, granuloma-like structures, PBMC, host-directed therapy, drug efficacy, preclinical models

## Abstract

Tuberculosis (TB) granulomas are complex multicellular structures central to disease pathogenesis and treatment challenges, notably due to bacterial persistence and immune modulation. Existing animal and *in vitro* models do not fully capture granuloma heterogeneity and immune complexity. Here, we review the biology and applications of granuloma-like structures (GLS) derived from human peripheral blood mononuclear cells infected with *Mycobacterium tuberculosis*, which recapitulate key features of early granuloma formation, including macrophage differentiation, lymphocyte involvement, and cytokine dynamics. Quantitative analysis identifies conserved bacterial growth trajectories and recurrent aggregate sizes, despite protocol diversity and donor variability. Furthermore, a minimal theoretical model illustrates how aggregate size may influence drug efficacy, through physical and biological mechanisms. Although underutilized in drug screening, GLS offer a promising intermediate platform by bridging simple cell monolayers to animal models, particularly for evaluating host-directed therapies. Standardized metrics and integration with computational models are needed to enhance reproducibility and translational relevance. GLS thus represent a valuable tool to investigate host–pathogen interactions and advance TB drug development under controlled immune conditions.

## Teaser

This review critically examines granuloma-like structures (GLS) from human peripheral blood mononuclear cells infected with *Mycobacterium tuberculosis*, analyzing their capacity to recapitulate early granuloma features, collecting the quantitative evidence of reproducible bacterial kinetics and GLS size, and assessing their current use for drug testing, while advocating for their standardization to bridge cell cultures and animal models in tuberculosis drug development and host-directed therapy.

## Introduction

Tuberculosis (TB), caused by *Mycobacterium tuberculosis* (*Mtb*), remains one of the leading infectious causes of death worldwide, despite the availability of effective antibiotic treatments (Global Tuberculosis Report 2024). Central to TB pathogenesis is the formation of granulomas, complex, multicellular structures that form in response to *Mtb* infection. These structures serve a protective role by containing bacilli, yet, paradoxically, they also provide a niche that favors their persistence (Cronan 2022, Rahlwes et al. 2023). Granuloma biology is marked by striking heterogeneity: their structure, cellular composition, and microenvironment vary considerably both between and within individuals, reflecting a spectrum of immunological responses and disease outcomes. In some instances, granulomas achieve bacterial clearance, while in others they progress to necrosis and tissue damage, thereby contributing to transmission and disease severity (Lin et al. 2006, Pai et al. 2016, Cadena et al. 2017, Russell et al. 2025). Importantly, these structures are also implicated in drug tolerance, as *Mtb* can persist in non-replicating or metabolically altered states within the granuloma core, shielded from host immunity and antibiotic activity (Gengenbacher and Kaufmann 2012).

Animal models, especially murine and non-human primates, have provided valuable insights into granuloma formation and host-pathogen interactions (Flynn 2006, Arrey et al. 2019); however, none fully reproduces the complexity of human TB. *In vitro* systems, such as macrophage monolayers or immortalized cell lines (*e.g*. THP-1), have yielded important mechanistic insights into intracellular *Mtb* growth and drug activity, but they lack immune complexity, as they exclude lymphocytes, dendritic cells, and the broader cytokine network, and fail to reproduce the spatial and temporal heterogeneity of granulomatous responses observed *in vivo* (Guirado and Schlesinger 2013, Esmail et al. 2014, Elkington et al. 2019). This gap is increasingly recognized as a bottleneck in TB drug development, particularly in the context of host-directed therapies (HDTs), which aim to modulate the immune response to improve treatment outcomes (Wallis and Hafner 2015, Zumla et al. 2016, Bielecka et al. 2017, Palucci and Delogu 2018, Jeong et al. 2022).

The growing interest in therapies targeting host–pathogen interaction (HPI) and immune-modulating adjuncts reinforces the need for experimental systems that capture not only bacterial clearance but also the influence of host cell states and immune activation on drug efficacy. However, limitations in throughput, cost, and ethical concerns also restrict their application for systematic drug testing (Elkington et al. 2019). In parallel, quantitative modeling approaches, including pharmacokinetic/pharmacodynamic and physiologically-based pharmacokinetic models, are being developed to integrate drug concentration dynamics with biological variability (Alffenaar et al. 2020, Karakitsios et al. 2025). These tools are now increasingly combined with *in silico* simulations of immune responses, employing ordinary differential equations and agent-based models to incorporate cellular behavior, spatial structure, and treatment timing into predictive frameworks (Segovia-Juarez et al. 2004, Cilfone et al. 2013, Dartois V 2014, Gideon et al. 2015). Integrating these approaches with biologically informed *in vitro* models may improve TB drug development, by treating the immune response as an active determinant of therapeutic outcome (Cubillos-Angulo et al. 2022).

In this context, granuloma-like structures (GLS) represent a promising *in vitro* model that integrates the experimental tractability of cell-based assays with some of the complex biological features of *in vivo* granulomas, notably their multicellular organization and structural complexity. Using donor-derived immune blood cells, these systems aim to reproduce early host–pathogen interactions under controlled conditions and allow investigation of the immune determinants of bacterial persistence.

Beyond traditional cell cultures, recent tissue engineering advances have introduced lung organoid and lung-on-a-chip platforms that successfully model TB by recapitulating tissue architecture, air-liquid interfaces, and biomechanical cues influencing host-pathogen interactions (Benam et al. 2015, Thacker et al. 2020). However, their implementation requires specialized fabrication and microfluidic expertise, which limits throughput for routine drug screening. In this context, GLS models serve as simplified organoid-like systems that retain multicellular and three-dimensional organization while remaining more accessible and scalable. The choice between these platforms ultimately depends on the specific research question and practical constraints of throughput and reproducibility

This review examines how current *in vitro* GLS models capture the biology of TB granulomas and assesses their potential and limitations as a quantitative platform for studying host–pathogen interactions and testing anti-TB therapies.

## Pathogenesis of TB and granuloma formation *in vivo*

### TB pathogenesis

TB is an airborne disease, where *Mtb* is transmitted in small droplets released by patients with active pulmonary infection and inhaled by susceptible hosts (Coleman et al. 2022, Warner et al. 2025). Upon reaching the lung alveoli, *Mtb* encounters resident alveolar macrophages (AMs), which represent the first line of cellular defense and, in most cases, efficiently eliminate the pathogen, achieving complete bacterial clearance. However, depending on factors such as exposure load, strain virulence and host susceptibility, *Mtb* may evade killing and replicate within infected and neighboring cells (Bastos et al. 2018). During the first two weeks following infection, *Mtb* replicates rapidly, disseminating to surrounding cells, including blood monocyte-derived interstitial macrophages (IMs). This process prompts inflammatory responses and the recruitment of additional immune cells to the primary infection site (Flynn and Chan 2001). Uncontrolled bacterial replication facilitates the further spread of *Mtb* via contiguous tissue invasion, as well as hematogenous and lymphatic dissemination, enabling colonization of distant tissues and organs (Bishai 2000, Cambier et al. 2014). Once adaptive immunity is established, T lymphocytes and other immune cells migrate toward the sites of bacterial replication, promoting granuloma formation (Fig. 1).

**Figure 1:**
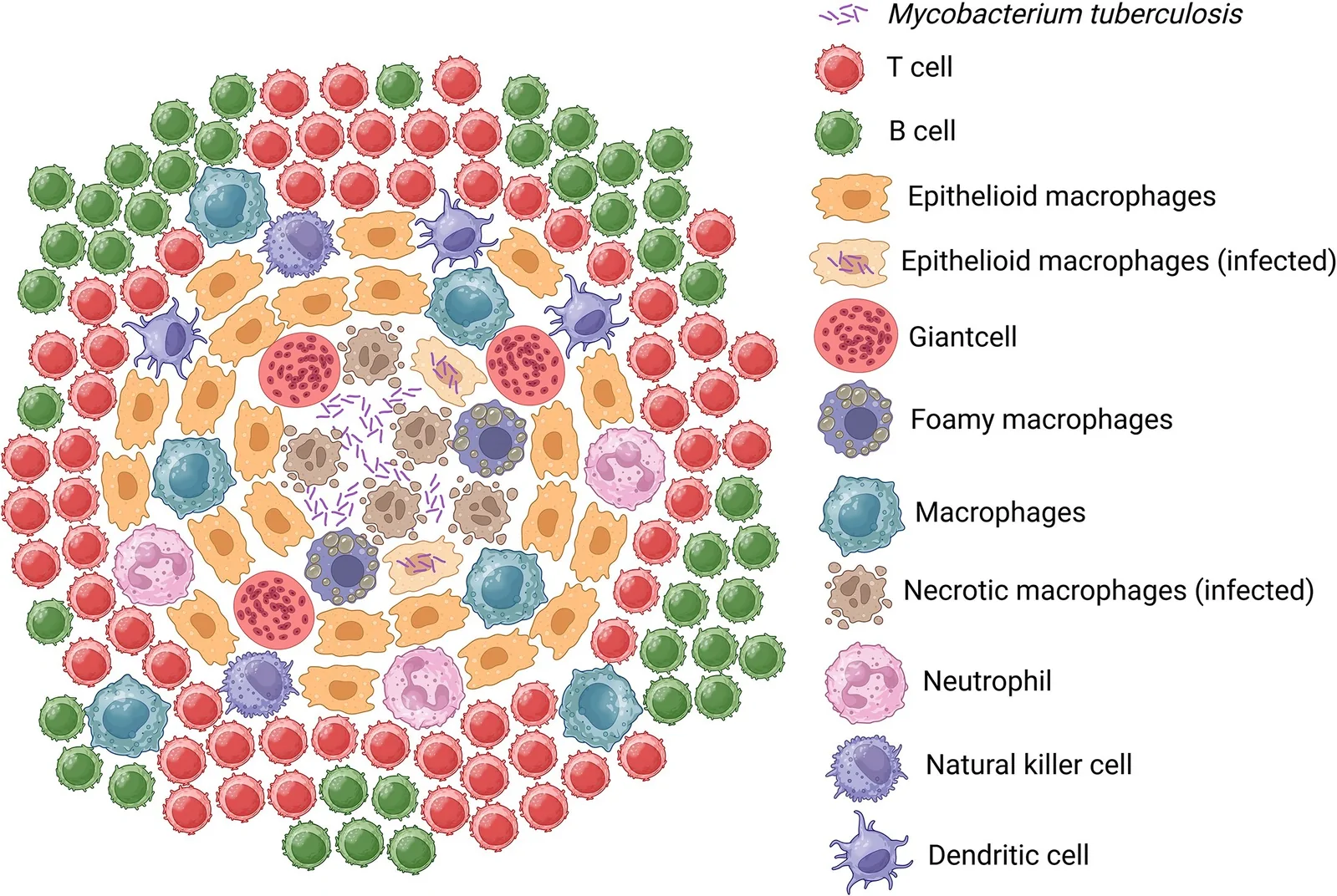
Schematic representation of a mature human tuberculous granuloma. A central core of infected and epithelioid macrophages, multinucleated giant cells and foamy macrophages surrounds necrotic material containing extracellular *Mycobacterium tuberculosis* (*Mtb*). This macrophage-rich core is enclosed by a peripheral cuff of lymphocytes, with T cells distributed throughout the cuff and B cells clustered in tertiary lymphoid-like aggregates at the outer edge; natural killer cells, neutrophils and dendritic cells are interspersed within the structure. Individual cell types are identified in the key. Created with BioRender.com.

Specifically, granulomas nucleate from aggregates of infected macrophages that progressively organize into multicellular structures (Chandra et al. 2022, Sawyer et al. 2023). Typically, they comprise a central core of macrophages, including epithelioid cells and multinucleated giant cells, surrounded by T and B lymphocytes, natural killer (NK) cells and neutrophils (Russell et al. 2025). The structure, composition, size, and histological features of each granuloma are dictated by local events that lead to different forms, ranging from calcified, solid, necrotic, or caseous granulomas. These distinct types of granulomas represent different outcomes of the complex host-pathogen interplay occurring at the tissue level.

The most likely outcome following *Mtb* infection is latent tuberculosis infection (LTBI), more recently termed TB infection, characterized by the presence of bacilli in host tissues without clinical signs or symptoms. Although direct experimental data describing the presence and metabolic state of *Mtb* during latency is lacking, indirect observations of *Mtb*-specific host immune response and bacterial biology, as well as evidence derived from animal models, support the existence of a long-term equilibrium between the pathogen and the host immune system (Gengenbacher and Kaufmann 2012). This dynamic interplay can persist for decades, maintained by *Mtb*-specific effector and memory T cells that contain bacterial replication and prevent pathological progression (Barry et al. 2009). Indeed, disruption of this balance—most frequently during the first 12–24 months post-infection, but potentially after much longer periods—results in granulomas with caseous or necrotic phenotypes. These lesions extend tissue damage, leading to lung cavitation and active disease. A revised conceptual framework on TB infection has been recently proposed, highlighting the fact that most of TB disease cases, at least in high-burden countries, result from recent or new *Mtb* infection (<2 years) rather than TB reactivation, somehow questioning the relevance of LTBI and *Mtb* dormancy on global TB burden (Dowdy and Behr 2022, Behr et al. 2024). Nevertheless, in low-burden countries most native active TB patients are older adults or the elderly, who are more likely to have been remotely infected, supporting the hypothesis of a long-term or even lifetime *Mtb* infection.

Amid the effective containment (LTBI) and overt disease (active TB) lies a spectrum of intermediate conditions that prove difficult to classify from both immunopathological and clinical perspectives (Goletti et al. 2022). Within this spectrum, “incipient TB” refers to cases likely to evolve to active disease in the near future, when host control of bacterial replication is being lost despite the absence of signs or symptoms. By contrast, “subclinical TB” lacks clinical manifestations but can be detected by radiological imaging and sometimes by microbiological detection of *Mtb*. These conditions highlight the complexity of TB pathogenesis, as the cellular and molecular mechanisms governing granuloma dynamics and tissue-level responses are still only partially understood (Drain et al. 2018).

### TB heterogeneity and the central role of granulomas

Elucidating the cellular and microenvironmental dynamics within the granuloma remains a central objective in TB research. Recent studies in animal models, combined with sophisticated techniques such as single-cell RNA sequencing, have begun to elucidate the complexity of *Mtb*–host interactions, as well as the cellular composition and dynamics of granulomas (Pisu et al. 2020). Early after infection, *Mtb* targets alveolar macrophages (CD11c+, SiglecF+), which are highly permissive to bacterial replication and display oxidative phosphorylation and fatty acid oxidation. Infected alveolar macrophages then migrate into the lung interstitium, where the infection spreads to recruited blood-derived monocytes that differentiate into interstitial macrophages. Unlike their alveolar counterparts, these upregulate glycolysis upon infection, show greater microbicidal activity and produce pro-inflammatory cytokines, adopting M1-like features. This functional divergence is also reflected in CD38 expression, which distinguishes permissive (CD38−) from controller (CD38+) macrophages (Pisu et al. 2020, 2024, Russell et al. 2025). Intracellular *Mtb*, and bacilli released extracellularly upon macrophage death, stimulate the secretion of chemokines and cytokines (IL-1, IL-8, CXCL-9/10) that attract monocytes, macrophages, neutrophils, and dendritic cells to the infection site (Kurashima et al. 1997, Ameixa and Friedland 2002, Silva Miranda et al. 2012), initiating the formation of the nascent granuloma. These immature granulomas are poorly effective in controlling bacterial replication, and bacilli are carried to the lung’s draining lymph nodes, where they prime adaptive immune responses.

The emergence of adaptive immunity promotes T- and B-cell migration towards the site of infection. Antigen-presenting cells release IL-12, promoting IFN-γ production by T cells and enhancing macrophages antimicrobial activity (Flynn et al. 1993, Cooper et al. 1997, Mayer‐Barber and Sher 2015). TNF-α, induced by IFN-γ and secreted by macrophages, dendritic cells and CD4 + T cells, contributes to granuloma maintenance but has a dual effect: while limiting bacterial growth, its excessive production can contribute to tissue damage and immunopathology (Dorhoi and Kaufmann 2014, Ravesloot-Chávez et al. 2021). IL-6 supports IFN-γ expression and cell survival (Ladel et al. 1997, Saunders et al. 2000), whereas anti-inflammatory cytokines such as IL-10 and TGF-β impair macrophage activation and may contribute to mycobacterial persistence (Rojas et al. 1999, O’Leary et al. 2011, Redford et al. 2011, Silva Miranda et al. 2012). The balance between these pro- and anti-inflammatory signals (Upadhyay et al. 2018) determines whether granulomas control bacterial growth or develop a permissive, tissue-damaging environment. This regulation is further shaped by the T-cell compartment itself: some *Mtb*-specific T cells may fail to penetrate the granuloma or acquire an exhausted phenotype through PD-1/PD-L1 or CTLA-4-dependent pathways, sustaining the immunosuppressive environment (Dorhoi and Kaufmann 2014, Russell et al. 2025). As granulomas mature (Cadena et al. 2017, Medrano et al. 2023), macrophages differentiate into foamy macrophages, epithelioid cells and multinucleated giant cells; in non-human primates, organized lesions can appear within 3–4 weeks.

Depending on the events taking place in each granuloma and the ability of the complex cellular milieu to combat *Mtb*, different granuloma types arise. These range from lesions that achieve complete killing of *Mtb*, where macrophages are replaced by fibroblast-like cells (resolved granulomas), to fibro-caseous granulomas, surrounded by a fibrotic cuff with peripheral T and B lymphocytes and characterized by a necrotic caseous centre rich in cellular debris and extracellular bacilli. Importantly, a single patient or infected animal may harbour a spectrum of different granulomas, reflecting the combined influence of the innate and adaptive immune responses and of the state and replication dynamics of the bacilli (Cadena et al. 2017, Lyu et al. 2024).

The physiological features of the bacilli within granulomas strongly influences bacterial eradication and tissue pathology. In permissive macrophages, *Mtb* often remains metabolically active and replicating, whereas in controller macrophages bacilli display signs of stress and fail to replicate, with intermediate states between these extremes also described (Russell et al. 2025). These states are associated with distinct transcriptional programs related to iron handling, central carbon metabolism, cell wall synthesis, growth, and responses to host-derived stress and reactive radicals, consistent with previous observations that *Mtb* can adopt dormant, non-replicating states in host tissues, with varying susceptibility to anti-TB drugs (Dartois V 2014). The heterogeneity of *Mtb* populations residing intracellularly, together with extracellular non-replicating bacilli in the caseum, represents a major challenge during TB therapy, as these stressed, non-replicating cells display relative drug tolerance and can persist despite multidrug treatment regimens.

## Experimental models of TB and granuloma-like structures

The significance of granuloma formation in TB is widely acknowledged, yet their study remains challenging, partly due to their inherent heterogeneity. Granulomas do not represent a uniform entity; rather, they encompass various manifestations that may or may not correlate with disease progression, adding complexity to their study (Pai et al. 2016, Sholeye et al. 2022). Additionally, no single animal model can fully replicate the diverse structures and compositions observed in human granulomas, as these differ significantly across species. Available models range from mice (Kramnik et al. 2000, Flynn 2006, Niazi et al. 2015, Arrey et al. 2019) to non-human primates (Walsh et al. 1996, Lin et al. 2006) to non-mammalian systems, such as the zebrafish (Volkman et al. 2010) and the nematode *Galleria mellonella* (Li et al. 2018), each offering unique advantages and limitations, and requiring careful selection according to the specific research question (Cronan 2022).

Despite their value for both basic and applied research, the use of *in vivo* models is largely constrained by ethical considerations, high costs, and time-consuming procedures. The development of increasingly sophisticated and accurate *in vitro* models of granuloma formation may help address these challenges and offer insights into the biology of human granulomas, particularly during their early formation stage. Such models may also prove useful for applied and translational research, offering a platform to evaluate efficacy of novel investigational anti-TB drugs. Indeed, inside granulomas, *Mtb* encounters a peculiar microenvironment characterized by limited nutrients and oxygen availability and reduced drug penetration, which cannot be fully reproduced in axenic cultures. *In vitro* granuloma models (Fig. 2) thus offer an attractive alternative, allowing for higher assay throughput, fewer ethical problems, and improved laboratory safety compared to *in vivo* assays.

**Figure 2:**
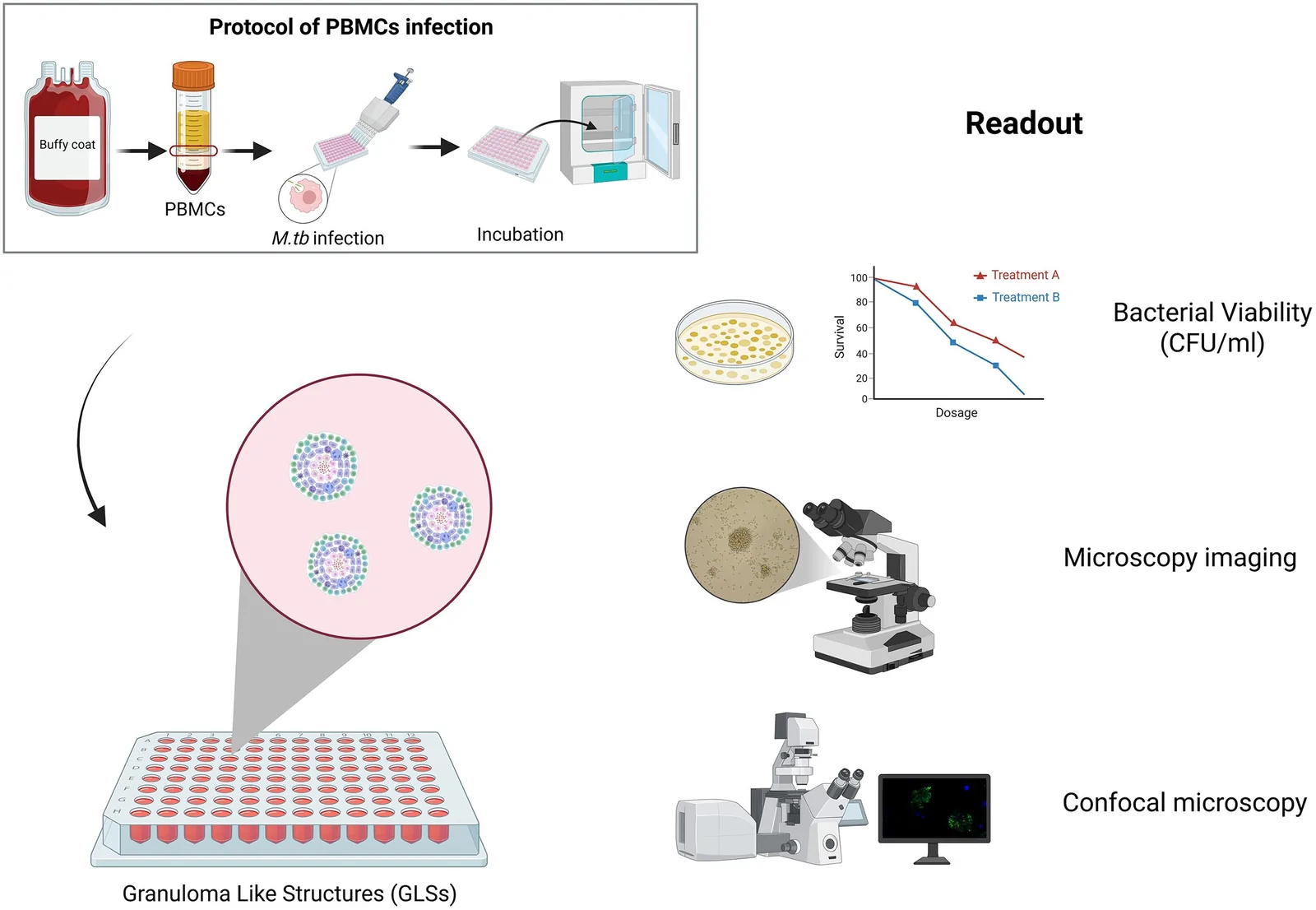
Schematic overview of a typical GLS assay. Peripheral blood mononuclear cells (PBMCs) are isolated from human donors and infected with *Mycobacterium tuberculosis* (*Mtb*) to promote the *in vitro* formation of three-dimensional cellular aggregates. Cells are seeded in multi-well plates, infected with *Mtb* and incubated for one to two weeks. The resulting GLS are then analysed through several readouts: colony-forming unit (CFU) enumeration for bacterial viability, brightfield microscopy for aggregate morphology, and confocal imaging for structural and cellular composition. Created with BioRender.com.

Although granulomas are inherently three-dimensional structures, *in vitro* models are conventionally classified as 2D or 3D based on whether cells are cultured on a flat surface or within a volumetric extracellular matrix.

The first two-dimensional (2-D) *in vitro* granuloma model was developed in 2004 (Puissegur et al. 2004) to investigate the molecular and cellular interactions occurring in the early stages of granuloma formation. In this dual system, granulomas were induced either by artificial beads coated with mycobacterial antigens—such as purified protein derivatives (PPD) or lipids—or by live mycobacteria. Within nine days post PBMC infection, this approach produced GLS resembling their *in vivo* counterparts in morphology and cellular differentiation. Activated macrophages, multinucleated giant cells, and epithelioid cells covered the bead surface, and were surrounded by a ring of lymphocyte-like cells.

Building on this, Guirado *et al*. developed an *in vitro* 2D PBMC-derived granuloma model, supplemented with autologous serum from either naïve or LTBI donors. This system allowed comparison of immune and mycobacterial determinants in individuals with different infection histories and demonstrated that patients with latent disease mounted significantly different granulomatous responses compared to naïve donors. Such observations confirmed the influence of immune memory on granuloma formation, mycobacterial survival, lymphocyte proliferation, cytokine balance, and lipid body accumulation (Guirado et al. 2015). Importantly, these findings underscore that a detailed understanding of the spatiotemporal dynamics of host immune responses in LTBI granulomas is essential for designing effective post-exposure therapies aimed at preventing progression to active TB (Rao et al. 2019).

Nevertheless, 2D models show several limitations, including the relatively low throughput, the difficulty of incorporating additional cell types, and the absence of an extracellular matrix, which is crucial for host cell biology and critical for regulating key processes in host-pathogen interaction, such as cell survival, phagolysosome fusion, autophagy, and cytokine secretion (Bielecka et al. 2017). An emerging paradigm in biology underscores that events in traditional 2D culture systems often differ substantially from those in three-dimensional (3D) settings (Benam et al. 2015). Since human disease occurs in 3D, and within the context of extracellular matrix, 2D systems cannot fully replicate *in vivo* conditions. By contrast, 3D *in vitro* models can better approximate the functional complexity of *in vivo* environments, while maintaining the same experimental tractability of traditional cell culture assays (Yamada and Cukierman 2007).

In 2013, Kapoor et al. established an innovative 3D biomimetic model by embedding PBMCs in a collagen matrix and infecting them with *Mtb* (Kapoor et al. 2013). This model recapitulated granulomas containing either dormant bacteria or actively replicating bacilli. After 8 days of infection, the bacilli exhibited hallmarks of dormancy—such as non-replicating persistence, rifampin tolerance, loss of acid-fastness, lipid body accumulation and transcriptional reprogramming—mimicking many features observed in human TB. Furthermore, treatment with anti-TNFα antibodies prompted bacterial resuscitation, as observed in patients. Although promising, this model has shown some limitations, such as low throughput, technical difficulties in maintaining a dynamic microenvironment over long time periods, and the need for collagenase digestion to recover cells for downstream analysis, such as CFU enumeration (Elkington et al. 2019).

The effect of an extracellular matrix was later tested directly. Arbués et al. compared PBMC-derived granulomas cultured with and without a collagen and fibronectin matrix, using the same donor cells, the same *Mtb* strain and the same multiplicity of infection (Arbués et al. 2021). Aggregate formation was favoured in the absence of the matrix, whereas its presence lowered the bacterial burden through an earlier induction of granulocyte-macrophage colony-stimulating factor, a cytokine that promotes macrophage survival. The matrix also created a hypoxic microenvironment in which the bacilli accumulated lipid inclusions and acquired a dormancy-like phenotype.

To better replicate *in vivo* conditions of human TB, Parasa et al. developed an *in vitro* lung tissue model consisting of a filter membrane scaffold and a collagen matrix supporting human fibroblasts and epithelial cell lines (Parasa et al. 2013). After tissue differentiation, the apical surface of the epithelial cells secreted mucus when exposed to air, mimicking anatomical and functional aspects of pulmonary tissue. Subsequently, upon introduction of *Mtb*-carrying macrophages, monocytes clustered around them to form granulomas. In this model, authors demonstrated that early granuloma formation is dependent on the virulence factor ESAT-6, which is secreted by the Type VII secretion system ESX-1, and that inhibition of matrix metalloproteinases, secreted by host cells in response to mycobacterial virulence factors, interferes with granuloma formation and reduces bacterial load.

Another innovative approach was developed by Tezera et al., who successfully reproduced the 3-D environment by incorporating virulent mycobacteria, PBMCs and collagen–alginate matrix into microspheres via bioelectrospray technology. This bioengineering platform incorporated a multiplex readout system, enabling simultaneous investigation of both host and pathogen responses and underscoring the critical role of the extracellular matrix in regulating infection (Tezera et al. 2017). Moreover, incorporating additional cell types into the microspheres further expanded the study of immune interactions. Despite its promising potential, implementation of this model is limited by the need for specialized equipment and technical expertise. A summary of the models described in this section is reported in Table 1.

**Table 1:** Key *in vitro* granuloma models discussed in Section 3. For each model the table summarises the spatial organisation of the aggregates, the use of an exogenous matrix, the main features and the principal experimental limitations.

Model	Spatial structure	Exogenous matrix	Main features	Limitations
Puissegur et al. (2004)	2D	No	Uses beads coated with mycobacterial antigens or live *M. bovis* BCG. Mimics human TB granulomas in morphology, cell recruitment and differentiation, and multinucleated giant cell formation.	Low throughput; limited inclusion of additional cell types; does not fully reproduce cellular physiology; uses BCG rather than *Mtb*.
Guirado et al. (2015)	2D	No	PBMCs from individuals with latent TB infection (LTBI) compared with naive donors. LTBI cultures aggregate earlier and control bacterial replication better, showing that host immune status shapes the granulomatous response.	Shares the inherent limitations of 2D models.
Arbués et al. (2021)	3D	Collagen + fibronectin	PBMCs embedded in a collagen and fibronectin ECM, compared directly against the same cells cultured in 2D. Aggregate formation is favoured in 2D, and the resulting aggregates are smaller than in 2D, whereas the ECM lowers bacterial burden through matrix-induced granulocyte-macrophage colony-stimulating factor, and the hypoxia drives a dormancy-like phenotype with lipid inclusions in the bacilli.	Requires collagenase digestion to recover bacteria.
Kapoor et al. (2013)	3D	Collagen (PureCol)	PBMCs embedded in a collagen matrix. Enables the study of *Mtb* dormancy and reactivation; bacilli develop rifampicin tolerance.	Maintenance of a dynamic microenvironment is difficult; requires collagenase digestion.
Parasa et al. (2013)	3D	Collagen	*Mtb*-infected macrophages introduced into an *in vitro* lung tissue model composed of stratified epithelium and fibroblasts capable of mucus secretion.	High complexity; low throughput; requires advanced technical expertise.
Tezera et al. (2017)	3D	Collagen + alginate	PBMC, *Mtb* and a collagen-alginate matrix incorporated into microspheres. Aggregate size is set by the encapsulation process rather than by cellular self-organisation.	Requires specialized equipment and advanced technical expertise.

ECM, extracellular matrix.

A recent study applied the PBMC-based 3D granuloma to 14 well-characterized clinical *Mtb* isolates representing five major phylogenetic lineages (L1–L5) to investigate the impact of mycobacterial genetic diversity on host–pathogen interactions within GLS (Arbués et al. 2025). This model discriminated strain-level differences in bacterial growth and granulomatous responses, extending its utility toward the investigation of pathogen diversity.

Beyond investigating granuloma formation and early host-pathogen interactions, GLS models may also support drug discovery. By reproducing aspects of the three-dimensional complexity of granulomas, which profoundly affects drug penetration and activity, they may improve the evaluation of investigational anti-TB drug candidates (Palucci et al. 2020). However, challenges remain, including the lack of high-throughput protocols, limited standardization for managing biological variability, and the technical complexity of performing GLS assays in a biosafety level 3 laboratory.

## Early stage of *in vitro* granuloma formation

In GLS models derived from PBMCs, the response relies on circulating monocytes rather than lung alveolar macrophages. Upon exposure to *Mtb*, these monocytes have been observed to differentiate into macrophages, even in the absence of exogenous cytokines or growth factors, consistent with monocyte-derived cultures, in which adhesion and pathogen stimulation induce a macrophage-like morphology (Birkness et al. 2007, Je et al. 2016). Infection appears to enhance this differentiation through pathogen recognition and endogenous signalling, although the extent of the process varies across donors and experimental setups (Palucci et al. 2020). When cultured as isolated macrophages, these cells permit *Mtb* replication but fail to organize into structured aggregates and lack phenotypic diversity; lipid-loaded foamy macrophages are rarely observed unless induced by lipid supplementation or prolonged culture (Peyron et al. 2008). Within PBMC cultures, lymphocytes are critical for both three-dimensional aggregates formation (Kapoor et al. 2013, Guirado et al. 2015), and macrophage differentiation into epithelioid cells and multinucleated giant cells (Birkness et al. 2007, Lay et al. 2007, Je et al. 2016).

Aggregate formation is accompanied by a pro-inflammatory phase dominated by TNF-α, IFN-γ, IL-6, IL-8, and IL-12, which typically peaks within the first 5–6 days post-infection (Birkness et al. 2007, Kapoor et al. 2013, Guirado et al. 2015, Je et al. 2016). This phase is associated with a predominantly M1-like macrophage phenotype (Huang et al. 2015) while TNF-α and IFN-γ levels correlate with GLS number and size (Agrawal et al. 2016). This M1-like state is further shaped by metabolic cues such as arginine and tryptophan availability (Zhang et al. 2024). As infection progresses, IL-10 increases after days 6–8, coinciding with a reduction in TNF-α and IFN-γ and a macrophage transition towards an M2-like phenotype (Guirado et al. 2015, Huang et al. 2015). Not all reports describe the same outcome: some identify a transition to an M2-dominated profile, while others report a stable, mixed M1/M2 balance persistent low-level inflammation and IL-10 secretion (Huang et al. 2015). These discrepancies may reflect donor variability, infection dose, or experimental conditions.

A LTBI background reinforces this response: lymphocytes from LTBI donors promote stronger GLS formation, higher cytokine production and bacterial control (Guirado et al. 2015, Crouser et al. 2017, Rao et al. 2019). The infecting strain also influences activation: across 14 clinical strains from five lineages, CD4 and CD8 T-cell activation correlated negatively with *Mtb* growth rate. Ancestral strains (L1, L5) elicited the strongest responses and modern Beijing strains (L2B, L2C) the weakest, independently of differences in bacterial burden (Arbués et al. 2025). Pre-existing immune experience, infection history and genetic background influence monocyte differentiation and contribute to donor-to-donor variability in GLS experiments (Palucci et al. 2020). The timing and magnitude of these dynamics shape the bacterial metabolic state and microenvironment, and therefore the interpretation of drug-efficacy experiments in GLS (Alonzi et al. 2024).

### Knowledge gaps: antigen-independent and innate-like lymphocyte functions

Since *Mtb*-specific memory lymphocytes are absent in unprimed donors, early GLS organization likely relies on innate-like rather than antigen-specific mechanisms. These pathways remain to be tested directly within the GLS system, but data from *in vivo* and *ex vivo* models provide a plausible framework to interpret this response.

First, naïve T cells can be activated in an antigen-independent manner through cytokine-driven bystander signalling or direct contact with monocytes and dendritic cells (Geginat et al. 2002, Lee et al. 2020, Sato et al. 2023). Toll-like receptors may provide additional co-stimulation, although this responsiveness is more pronounced in memory T cells.

Second, this early response may be supported by non-classical T-cell populations that are innately reactive by nature: γδ T cells, which recognize non-peptide antigens and target infected macrophages (Silva Miranda et al. 2012, Ravesloot-Chávez et al. 2021); MAIT cells, which respond to microbial vitamin-B metabolites (Napier et al. 2015, Meermeier et al. 2022); and iNKT cells, which recognize glycolipid antigens (Gao and Williams 2015), with CD161 expression associated with innate-like reactivity across these subsets. The representation of these innate-like lymphocytes in PBMC cultures varies: γδ T cells and NK cells are typically retained, whereas MAIT cells often appear underrepresented.

### Limitations

Despite these correspondences, GLS capture only the early, innate phase of the host response, before antigen-specific adaptive immunity is established, which means the resulting aggregates remain structurally simpler than mature granulomas *in vivo*. Over a typical two-week culture, GLS do not develop compact organization or a necrotic caseous core, and neutrophils and antigen-experienced T- and B-cell responses remain underrepresented or absent. Certain microenvironmental features can nonetheless be engineered, for instance through extracellular-matrix–based systems that reproduce hypoxia (Arbués et al. 2021). This early-stage focus, however, should not be seen only as a limitation: because the initial host-*Mtb* encounter is difficult to access *in vivo*, GLS offer an approachable human-cell system to isolate, manipulate and dissect these early interactions, and a practical platform for testing anti-TB drugs and host-directed therapies.

## *In vitro* models to assess drug efficacy

*In vitro* platforms for anti-tuberculosis drug screening are central to early-stage drug discovery. Traditional time-kill and MIC assays measure the activity of a compound against rapidly replicating *Mtb* in nutrient-rich axenic broths, and therefore report intrinsic potency under simplified conditions.

In the host, the efficacy of that compound depends on determinants these assays do not reproduce. Bacilli within infected macrophages range from actively replicating to stressed and non-replicating (Russell et al. 2025), and non-replicating bacilli tolerate drugs directed against processes required for growth (Gold and Nathan 2017); moreover, immune activation of the host cell can itself induce drug tolerance in intracellular *Mtb*, independently of replication rate (Liu et al. 2016).

The same discrepancy affects screening itself: since almost all current TB drugs were identified in axenic media, compounds may be selected whose targets are essential for growth in broth but not in the conditions encountered *in vivo*, and no standard screening condition modelling host microenvironments has been established (Yuan and Sampson 2018). Efficacy further depends on how much compound reaches the bacterium, which is governed by drug disposition in tissues (Dartois 2014, Karakitsios et al. 2025). Two additional factors act instead on the measurement: immune host cells contribute to bacterial killing independently of the compound, and compounds may be cytotoxic to those cells, altering both the bacterial burden and the readout used to quantify it.

To achieve reproducible and scalable workflows, preclinical screening often relies on macrophage monolayer models, particularly immortalized cell lines such as THP-1 (Shah et al. 2022, Gries et al. 2024). THP-1 cells exhibit phenotypic and functional characteristics similar to those of primary human macrophages, allowing identification of compounds that target intracellular bacterial replication or host pathways crucial for infection and survival. THP-1–based assays have yielded data on the intracellular efficacy of first- and second-line TB drugs. Rifampin demonstrated the highest potency (Chanwong et al. 2007); isoniazid and moxifloxacin also showed strong activity, followed by linezolid and levofloxacin; ethambutol and ofloxacin were moderately effective (Rey-Jurado et al. 2013), while pyrazinamide showed no effect on intracellular *Mtb* growth, consistent with its role against non-replicating bacteria (Heifets et al. 2000). However, potency values returned by such assays depend on the host cell employed: *Mtb* is approximately 100-fold less susceptible to rifampin within hypoxic, lipid-loaded macrophages than within normoxic THP-1–derived macrophages, and a systematic comparison of anti-TB drug potency between primary macrophages and immortalized lines is still lacking, partly because cell lines differ from primary resting macrophages not only in immune diversity but also in their basal metabolic and transcriptomic states (Dartois and Rubin 2022). Monolayers also lack the three-dimensional organization and the broader signalling networks of a multicellular environment.

A limited number of studies have measured drug susceptibility in three-dimensional systems, with variable results (Table 2). Using automated high-content imaging of PBMC-derived aggregates, Silva-Miranda et al. (2015) compared MIC_50_ values across extracellular, single-macrophage, and *in vitro* granuloma assays: isoniazid potency remained stable, whereas moxifloxacin and linezolid exhibited a 10- to 15-fold loss of potency within the aggregate, which the authors attributed to poor physical permeability; conversely, pyrazinamide—devoid of activity against extracellular bacilli—displayed measurable activity within the granuloma (MIC_50_ ≈ 101 µg/ml). This effect was more pronounced in the microsphere model: pyrazinamide, inactive in 7H9 broth and in 2-D culture, cleared *Mtb* within the spheres, and isoniazid was more bactericidal in three dimensions than in monolayer, coinciding with the upregulation of mycobacterial stress genes (Bielecka et al. 2017). Both observations are consistent with previous finding that collagen-embedded PBMCs promote a dormant, rifampin-tolerant state (Kapoor et al. 2013): the three-dimensional format alters the physiological state of the bacilli, and not only the access of the compound—a dependence formalized quantitatively in Section 6.2. The two platforms are not equivalent in this respect, since the extracellular matrix present in microspheres itself regulates host cell survival, phagolysosome fusion, autophagy and cytokine secretion (Section 3). The same platform was subsequently used to compare clofazimine, three carbapenems and nitazoxanide, clofazimine proving the most effective (Gonzalo et al. 2022), and to examine strain-dependent differences in treatment response, the laboratory strain H37Rv appearing attenuated and more antibiotic-susceptible than clinical isolates (Bielecka et al. 2024).

**Table 2:** Studies evaluating drug efficacy in human three-dimensional or PBMC-based infection models. For each study the table reports the culture system, the cells and mycobacterial strain used, the compounds tested, and the main efficacy outcome.

Study	System	Exogenous matrix	Cells/strain	Drug addition	Readout	Duration	Compounds	Key efficacy outcome
Silva-Miranda et al. (2015)	PBMC-derived aggregates	No	PBMC; H37Rv-GFP	Day 3, after aggregate formation	Automated high-content imaging; GFP spot counting; aggregate number	5 days exposure	INH, MFX, LZD, PZA	INH potency stable; 10–15-fold loss of potency for MFX and LZD within aggregates; PZA, inactive extracellularly, showed measurable intragranuloma activity
Palucci et al. (2020)	PBMC-derived GLS	No	PBMC; H37Rv and clinical strain H3 (MOI 1:1)	Day 3 post-infection	CFU; GLS number and size	Up to 12 days total culture	INH; cysteamine, cystamine; capreomycin; amikacin	Cysteamine/cystamine reduced GLS number and *Mtb* CFU; INH alone reduced GLS number. Amikacin synergized with cysteamine/cystamine, whereas capreomycin was less effective and non-additive
Palucci et al. (2023)	PBMC-derived GLS	No	PBMC; *Mycobacterium abscessus*	Day 3 post-infection	CFU; GLS number and size	Up to day 12 post-infection	Cysteamine, cystamine ± amikacin	Enhanced efficacy of combinatorial host-directed treatments (cysteamine/cystamine ± amikacin) on both intracellular bacterial load and aggregate stability.
Bianco et al. (2023)	PBMC infection model (not GLS)	No	PBMCs; H37Rv and clinical isolates GM2659, *Mtb*H3, *Mtb*EAI	Day 4 post-infection	Intracellular CFU	CFU at day 8 or 12 post-infection	Everolimus (HDT)	Everolimus did not significantly reduce *Mtb* growth, concordant with the negative phase II adjunctive trial.
Bielecka et al. (2017)	PBMC in bioelectrospray microspheres	Alginate + type I collagen	PBMC; H37Rv-lux (MOI 0.1)	Day 1 or day 6	Longitudinal luminescence, validated against CFU	Longitudinal monitoring	RIF, INH, EMB, PZA, D-cycloserine, MFX, LZD; microfluidic PK	INH more active than in 2D, and PZA fully active, coinciding with matrix-induced bacterial stress and dormancy.
Gonzalo et al. (2022)	PBMC in bioelectrospray microspheres	Alginate + collagen	PBMC; H37Rv-lux	At microsphere generation	Luminescence	≥ 21 days exposure	Clofazimine; three carbapenems + clavulanate; nitazoxanide	Clofazimine proved the most bactericidal; carbapenems (with clavulanate) showed modest activity and nitazoxanide only at high concentrations.
Bielecka et al. (2024)	PBMC in bioelectrospray microspheres	Alginate + collagen	PBMC; H37Rv and clinical isolates, including MDR	Day 1	Luminescence; host gene expression	14 to 21 days	Verapamil with bedaquiline or delamanid	H37Rv appeared attenuated and more drug-susceptible than clinical isolates in 3D; adjunctive verapamil significantly boosted bedaquiline and delamanid efficacy.

Drug addition, time point at which compounds were added relative to infection. Duration, total assay length or exposure window, as reported. INH, isoniazid; MFX, moxifloxacin; LZD, linezolid; PZA, pyrazinamide; RIF, rifampicin; EMB, ethambutol; HDT, host-directed therapy; PK, pharmacokinetics.

Within PBMC-derived GLS, only a limited number of studies have directly evaluated antimicrobial efficacy, focusing primarily on multi-drug regimens and non-tuberculous mycobacteria. Palucci et al. (2020) reported that cysteamine and cystamine reduced both the number of GLS and the intracellular colony forming units (CFUs), with isoniazid alone also lowering the number of GLS. Combined with these transglutaminase-2 inhibitors, amikacin produced an enhanced restriction of the intracellular bacilli, whereas capreomycin—less effective than the inhibitors on its own—added no further benefit. To ensure methodological robustness, these assays evaluated drug effects by simultaneously measuring bacterial burden and structural parameters, reporting a reduction in total GLS counts while the average size of the remaining aggregates remained unchanged. This evaluation framework was later extended to *Mycobacterium abscessus* (Palucci et al. 2023), confirming the enhanced efficacy of combinatorial treatments on both bacterial load and aggregate counts in a multi-cellular context.

In addition to testing direct antimicrobials, multicellular PBMC-based models provide a valuable platform for evaluating host-directed therapies (HDTs), which aim to adjunctively boost host immune responses, modulate inflammation, or optimize drug delivery (Guirado and Schlesinger 2013), Palucci and Delogu 2018). Although primary cell assays are sensitive to pre-analytical factors such as cryopreservation, storage duration and sample processing, they have been used to evaluate clinical-stage candidates. For example, Bianco et al. (2023) evaluated everolimus, a clinical-stage HDT candidate, in a PBMC infection model; despite inducing host autophagy and ROS production, it did not significantly reduce *Mtb* growth, consistent with the limited microbiological benefit reported in a phase II adjunctive trial.

A recurring caveat is that apparent compound potency depends on several experimental variables: host cell characteristics, assay format, timing of drug addition, and donor immune background [(Palucci et al. 2020), Table 2]. Combined with the logistical demands of long-term biosafety level 3 (BSL-3) culture, this variability makes reported efficacies difficult to compare across laboratories, which constitutes a major obstacle to the reproducible, standardized use of these systems and, ultimately, to their broader adoption in anti-TB drug testing and development.

## Quantitative exploration of GLS assays

This Section addresses whether reproducible and generalizable quantitative parameters can be identified to support the development of standardized drug testing pipelines, potentially positioning GLS as an informative *in vitro* translational platform.

Several factors challenge the routine adoption of GLS for pharmacological screening. Beyond the donor-derived variability and logistical constraints already discussed, the complexity of the system, combined with heterogeneity in aggregate formation and bacterial replication, limits its operational standardization and, by extension, its integration into drug discovery pipelines. However, despite methodological diversity, many GLS protocols converge on a core objective: characterizing *Mtb*-induced aggregate formation in a controlled environment (Fig. 2).

Analyses of GLS commonly rely on multiple experimental readouts—CFU enumeration, aggregate morphology, cytokine secretion, and cellular composition—that are measured and interpreted independently (Figs. 2 and 3). This conventional approach emphasizes single-parameter evaluation and does not explicitly capture the interconnected nature of bacterial burden, host immune responses, and structural organization within the GLS. Moving toward an integrated analytical framework (Fig. 3) would enable the combination of multiple GLS readouts into system-level descriptors, facilitating comparison across studies and supporting quantitative interpretation through computational modeling.

**Figure 3:**
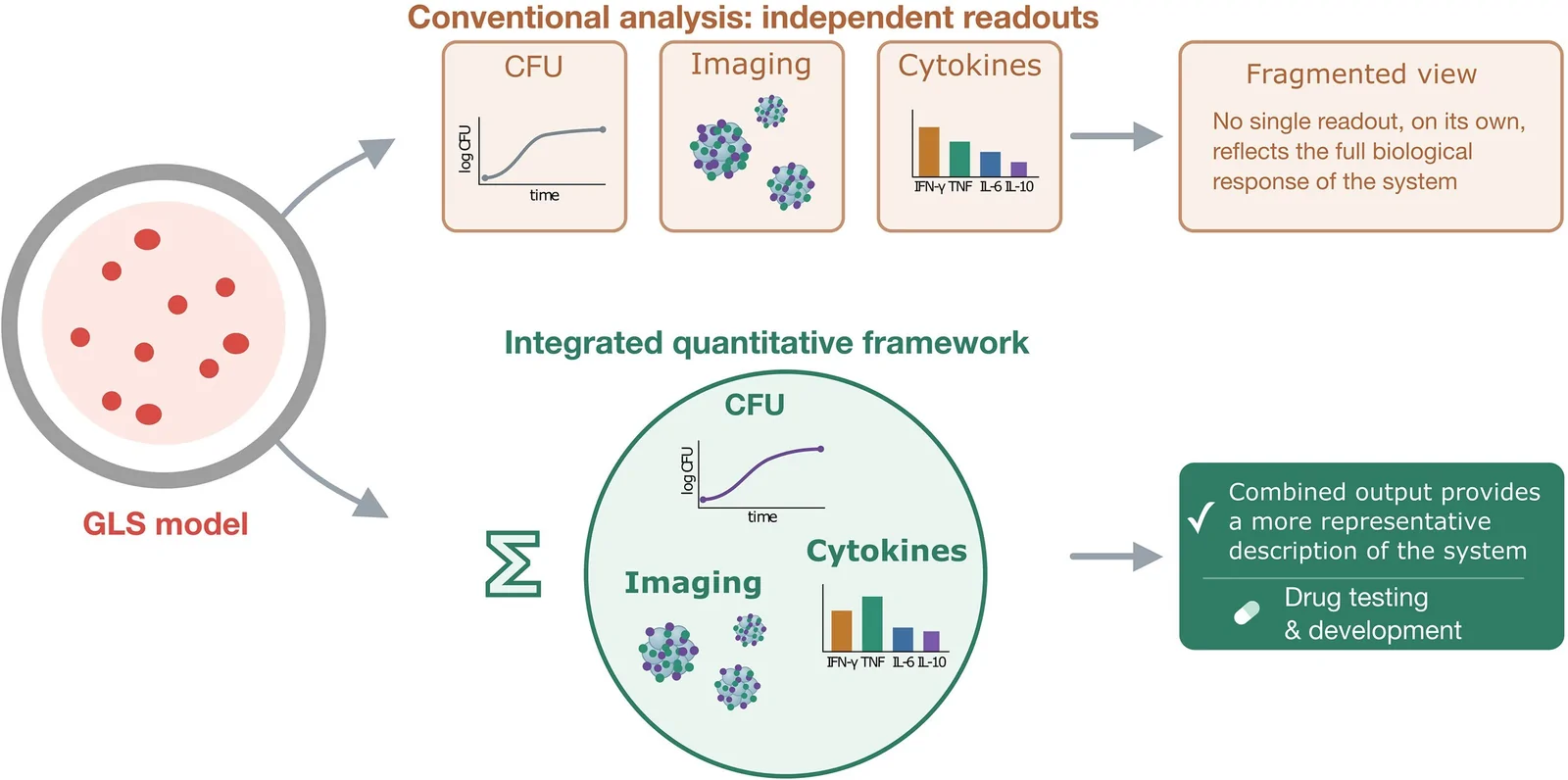
From independent readouts to integrated quantitative descriptors of GLS. (Top row) In conventional GLS analyses, experimental outcomes such as colony-forming units (CFU), aggregate size, and cytokine levels are quantified as independent readouts and interpreted separately. While informative, this approach does not capture the interdependence between bacterial burden, host immune response, and structural organisation within the aggregate. (Bottom row) In an integrated framework, the same measurements are combined into system-level descriptors that represent the functional state of the structure. Such descriptors allow drug efficacy and experimental variability to be assessed quantitatively, and provide a direct interface with *in silico* and mathematical modelling.

Focusing on PBMC-based models infected with virulent *Mtb* strains, and accounting for relevant experimental variations, might allow the identification of recurring quantitative descriptors across systems. Thus, comparative analyses could examine whether such patterns can be discerned and codified to support a future framework for standardized GLS assays.

### Quantitative metrics

GLS systems have been applied to a range of experimental purposes, from specific mechanistic inquiries, such as macrophage polarization or dendritic cell dynamics (Lay et al. 2007, Huang et al. 2015), to broader attempts at reproducing *in vivo* granuloma architectures. While these studies have predominantly emphasized qualitative immunological insights, rigorous quantitative analysis remains comparatively limited, hindering reproducibility and the evaluation of GLS in pharmacological contexts.

Data commonly reported in the literature fall into two broad categories: (*i*) CFU counts that provide robust quantitative information on bacterial burden and (*ii*) microscopy imaging predominantly used qualitatively to confirm granuloma-like aggregate formation (Fig. 2). Bacterial burden itself is not always quantified in the same way: while CFU enumeration is the most common approach, some studies rely on fluorescent (GFP) or bioluminescent reporter strains, and drug-testing studies may report only MIC values, so that the resulting measures are not directly comparable (Silva-Miranda et al. 2015, Bielecka et al. 2017). Quantitative descriptors derived from selected images, such as the size of individual structures, are rarely collected systematically, which prevents comprehensive and reproducible evaluation of aggregate morphology across experiments.

CFU enumeration, when available as longitudinal data, capture bacterial replication under varying immune conditions, while aggregate imaging reflects the structural outcome of host-pathogen interactions. Yet these outputs are often reported independently, without coordinated temporal analysis, being CFU data frequently limited to endpoint measurements. Likewise, imaging data are typically limited to a few selected fields, precluding comprehensive and statistically reliable assessments of aggregate morphology.

We analysed the most representative set of GLS studies (Table 3). Quantitative and semi-quantitative data relevant to bacterial load, aggregate morphology, cellular composition (Supplementary data A-Table S1), and cytokine profiles were extracted to facilitate comparison. The focus was restricted to PBMC-derived models infected with virulent *Mtb* strains to ensure comparability and to highlight both inter-study variability and recurring patterns that may serve as a basis for standardization. Four key parameters were taken into consideration:

- *CFU measurements*. Comparison across studies revealed a reproducible growth trajectory despite variations in key experimental parameters such as Multiplicity of Infection (MOI), which ranged from 1:200 to 1:1 (Fig. 4A, Supplementary data A-Table S2). A consistent pattern emerged: an initial exponential phase typically followed by growth deceleration, with final bacterial load varying proportionally with the initial bacterial load and inversely with the MOI. Because studies report bacterial load in different units, trajectories are compared as the fold increase relative to the first measurement. On this scale, growth at the end of culture ranged from about 6 to 60 fold, while the MOI varied two hundred fold. Trajectory conservation despite differing initial conditions suggests a robust biological behavior that could serve as a baseline for assessing antimicrobial efficacy in a standardized framework. Across fourteen clinical isolates cultured in the same system by the same laboratory, the growth rate between day 1 and day 8 ranged from 8 to 136 (Arbués et al. 2025).
- *Aggregate size*. Albeit inconsistently, aggregate size is often reported as an inherent validation of the *in vitro* model. Stable structure sizes generally ranged between 100–200 μm, though outcomes varied due to several factors, such as MOI (Fig. 4B, Supplementary data A-Table S3). High MOI typically resulted in numerous smaller agglomerates that may subsequently merge, whereas insufficient, low-MOI fail to produce stable GLS. At both extremes aggregates may fail to form altogether, and the values at which this occurs differ between systems (Seitzer and Gerdes 2003, Kapoor et al. 2013). These patterns suggest that the formation of larger granulomas may require extended culture periods and distinct cellular activation or differentiation processes. The outcome may also be influenced by the culture format, from well-size limitations to the presence of an exogenous matrix: with the same donor cells, strain and MOI, aggregates reached about 200 μm without a matrix and about 100 μm when the cells were embedded in one (Arbués et al. 2021).
- *Cytokine profiles*. These were less consistently reported, potentially hindering a comprehensive understanding of GLS models. Only three independent studies provided limited quantitative information, which is often highly specific or context dependent (Birkness et al. 2007, Guirado et al. 2015, Huang et al. 2015) (Fig. 4C and D, Supplementary data A-Table S4). Although TNF-α and IFN-γ are repeatedly described as critical for granuloma formation and maintenance, precise quantitative information remains limited and thus restricts accurate interpretation, underscoring the need for their systematic profiling in future GLS studies.
- *Spatial distribution and abundance*. Information on GLS spatial distribution within wells is also scarce. Although aggregate size can often be estimated from published images, data on the number of GLS per well or the percentage of surface area they occupy are rarely provided, and the few available come from systems with different settings (Supplementary data A-Table S5). The scarcity of such metrics represents a significant limitation, as they are essential for translating *in vitro* observations to physiologically relevant scales and for understanding infection dynamics and drug diffusion properties.

**Figure 4:**
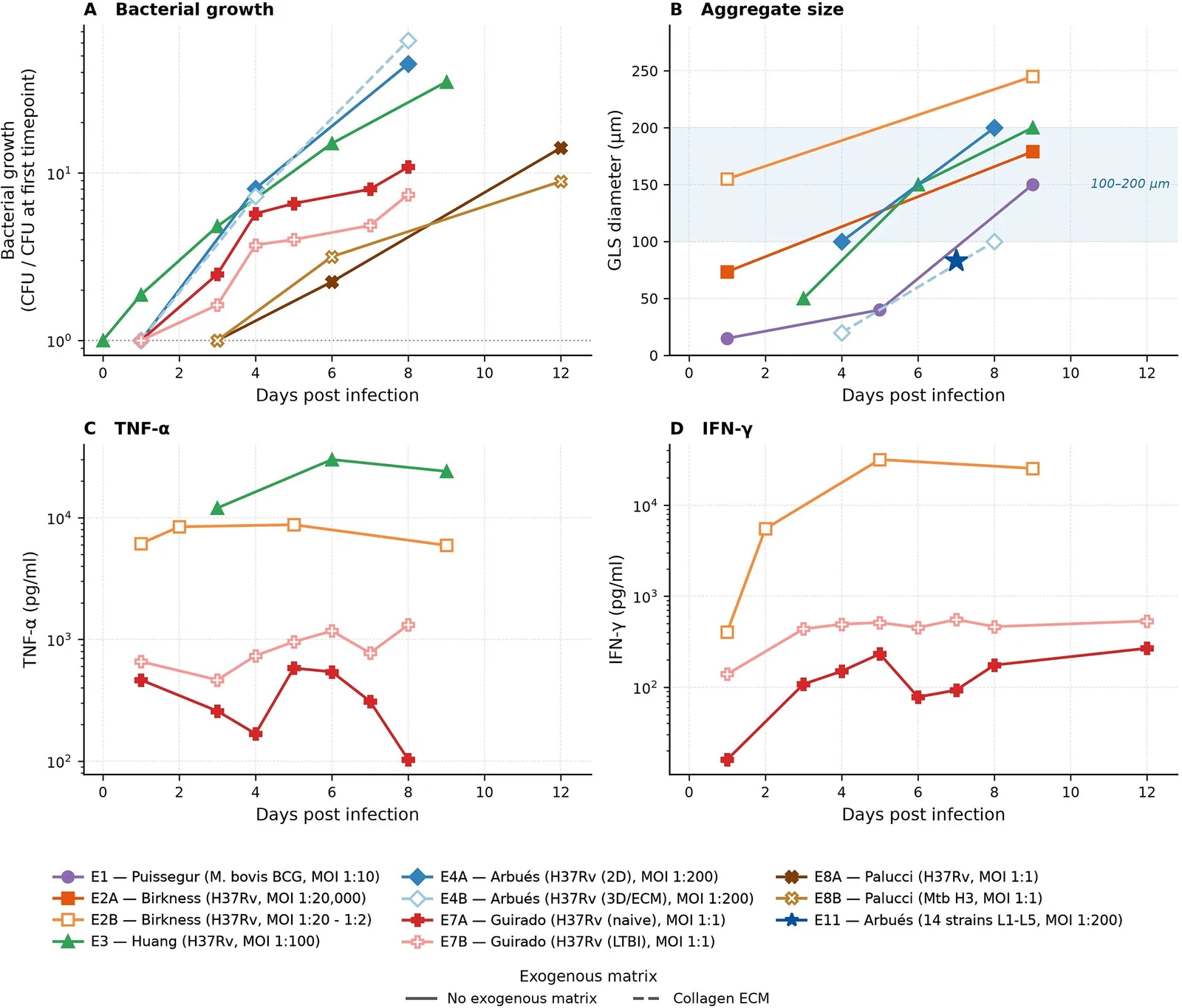
Quantitative readouts of GLS assays. All four panels share the same *x*-axis (days post infection). Colour identifies the study and line style indicates whether an exogenous extracellular matrix was used. Experiment identifiers (E1–E12) are those of Table 3.(A) Bacterial growth, expressed as the fold increase in colony-forming units (CFU) relative to the first measurement of each study (Supplementary Table S2. (B) Aggregate diameter. The shaded band marks the 100–200 µm range that recurs across studies (Supplementary Table S3). (C, D) Concentrations of tumour necrosis factor alpha (TNF-α) and interferon gamma (IFN-γ) in culture supernatants (Supplementary Table S4).

**Table 3:** Experimental design and quantitative readouts of the GLS studies analysed in Section 6. For each study (E-identifier) the table reports the culture conditions, the mycobacterial strain and infection parameters, and the main quantitative readouts—GLS diameter, GLS number, and CFU growth.

ID	Ref.	Culture surface	Exogenous matrix	Strain	Cells	MOI	Incubation	Infection procedure	GLS diameter	GLS number	CFU growth	Other readouts	Notes
**E1**	Puissegur et al. (2004)	Adherent plate	No	*M. bovis* BCG	10^6 PBMC/well	1:10 (BCG:cells); inoculum 10^5 (D)	15 d	Plated, then infected; no wash reported	150 (d9)	N/R	N/R	—	Diameter estimated from images
**E2A**	Birkness et al. (2007)	Non-adherent surface	No	H37Rv	2 × 10^5 PBMC + 2 × 10^5 autologous Mf/well	1:20 000 (*Mtb*:Mf); inoculum 10^1 (D)	9 d	Combined at plating; no wash reported	179 ± 37 (d9)	N/R	N/R	Cytokines	—
**E2B**	Birkness et al. (2007)	Non-adherent surface	No	H37Rv	2 × 10^5 PBMC + 2 × 10^5 autologous Mf/well	1:20–1:2 (*Mtb*:Mf); inoculum 10^4–10^5 (D)	9 d	Combined at plating; no wash reported	245 ± 64 (d9)	N/R	N/R	Cytokines	—
**E3**	Huang et al. (2015)	Adherent plate	No	H37Rv	10^6 PBMC/well	1:100 (*Mtb*:cells)	9 d	Plated, then infected; no wash reported	200 (d9)	5.05 (d6)	34 (d0-d9)	Cytokines	Diameter estimated from images; values are the untreated control
**E4A**	Arbués et al. (2021)	Adherent plate	No	H37Rv	2.5 × 10^6 PBMC/ml	1:200 (*Mtb*:PBMC)	8 d	Infected, then plated; no wash reported	200 (d8)	10–15 (d8)	44 (d1-d8)	—	Diameter and number estimated from images
**E4B**	Arbués et al. (2021)	Embedded in ECM	Collagen + fibronectin	H37Rv	2.5 × 10^6 PBMC/ml	1:200 (*Mtb*:PBMC)	8 d	Infected, then plated and embedded in ECM; no wash reported	100 (d8)	8 (d8)	61 (d1-d8)	—	Diameter and number estimated from images
**E5A**	Seitzer and Gerdes (2003)	Non-adherent surface (agarose-coated)	No	H37Rv	6 × 10^5 PBMC/well	1:150, 1:85, 1:42, 1:4	11 d	Co-incubated from the start; no wash reported	589 ± 38 (d7)	1.2 ± 0.6 (d4)	N/R	—	Size values are from uninfected spheroids
**E5B**	Seitzer and Gerdes (2003)	Non-adherent surface (agarose-coated)	No	*M. bovis* BCG	6 × 10^5 PBMC/well	1:150, 1:85, 1:42, 1:4	11 d	Co-incubated from the start; no wash reported	589 ± 38 (d7)	1.2 ± 0.6 (d4)	N/R	—	Size values are from uninfected spheroids
**E6**	Lay et al. (2007)	Adherent plate	No	H37Rv, Erdman, *M. microti*, Beijing	10^6 PBMC/well	1:1000 (*Mtb*:cells); 1:5000 (beads:cells)	30 d	Plated, then infected with bacteria or antigen-coated beads; no wash reported	N/R	N/R	N/R	Cell numbers and proportions	—
**E7A**	Guirado et al. (2015)	Adherent plate	No	H37Rv (naive donors)	2 × 10^6 PBMC/ml (2 × 10^5 monocytes)	1:1 (*Mtb*:Mf)	12 d	Infected immediately after isolation; no wash reported; medium and serum replaced every 4 d	N/R	N/R	9.9 (d1-d8)	Cytokines	—
**E7B**	Guirado et al. (2015)	Adherent plate	No	H37Rv (LTBI donors)	2 × 10^6 PBMC/ml (2 × 10^5 monocytes)	1:1 (*Mtb*:Mf)	12 d	Infected immediately after isolation; no wash reported; medium and serum replaced every 4 d	N/R	N/R	6.4 (d1-d8)	Cytokines	—
**E8A**	Palucci et al. (2020)	Adherent plate	No	H37Rv	PBMC (∼1 × 10^5 monocytes)	1:1 (*Mtb*:monocytes)	12 d	Infected immediately after isolation; no wash reported	∼126 (d10)	8 (d10)	13 (d3-d12)	—	—
**E8B**	Palucci et al. (2020)	Adherent plate	No	*Mtb* H3 (clinical isolate)	PBMC (∼1 × 10^5 monocytes)	1:1 (*Mtb*:monocytes)	12 d	Infected immediately after isolation; no wash reported	∼107 (d10)	5 (d10)	7.9 (d3-d12)	—	—
**E9**	Palucci et al. (2023)	Adherent plate	No	*M. abscessus* MAB-S/MAB-R	PBMC (∼1 × 10^5 monocytes)	MAB-S 1:1; MAB-R 1:10 (*Mtb*:monocytes)	12 d	Infected immediately after isolation; no wash reported	∼94/93 (d10)	30/35 (d10)	n.a. (single endpoint, d12)	Cytokines	—
**E10**	Kapoor et al. (2013)	Embedded in ECM	Collagen (PureCol)	H37Rv	5 × 10^5 PBMC/well	1:0.1 (*Mtb*:Mf)	14 d	Mixed with ECM, then infected within the matrix	N/R	N/R	N/R	—	—
**E11**	Arbués et al. (2025)	Embedded in ECM	Collagen + fibronectin	14 clinical strains (lineages L1-L5)	1.25 × 10^6 PBMC/well	1:200 (*Mtb*:PBMC)	8 d	Infected, then plated and embedded in ECM	66–100 (d7)	22 (d7)	8.1–136 (d1-d8)	Aspect ratio, aggregate formation score	Range across 14 clinical strains
**E12**	Silva-Miranda et al. (2015)	Adherent plate	No	H37Rv-GFP	3.5 × 10^5 PBMC/well	Not reported	8 d (3 d formation + 5 d exposure)	Infected 1 h in suspension, washed, then plated	N/R	N/R	N/R	—	Bacterial readout by GFP imaging

ID, identifier used in the text, figures and Supplementary data. Culture surface, substrate on which the cells were grown. Exogenous matrix, whether cells were embedded in a hydrogel. Cells, source and number of cells seeded per well or per millilitre, as stated in the original study. MOI, bacteria per cell, as reported or derived from the stated inoculum; (D) marks a derived value. Incubation, total culture duration. Infection procedure, reported in order: whether cells were plated before or after infection, whether they were washed after infection, and whether cells or medium were added during culture. GLS diameter (µm) and GLS number (aggregates per field), last value reported by the study, with the day of measurement in brackets; full time courses are given in Supplementary data A. CFU growth, relative change in bacterial load between the first and last available time point, (CFU_final − CFU_initial)/CFU_initial, over the time window given in brackets; being dimensionless, it is comparable across studies that used different inocula and reporting units. Other readouts, further quantitative data in Supplementary data A. Mf, macrophages. N/R, not reported in that study.

Among the various readouts, CFU counts and aggregate size distributions emerged as particularly informative. When considered together, these two metrics provide a more comprehensive picture of the experimental outcome than either does individually: CFU counts do not confirm GLS formation or maturation, whereas aggregate size alone does not capture bacterial replication. Both are likely to influence drug penetration and efficacy (Section 6.2), and their systematic recording would enhance the accuracy and reliability of pharmacological evaluations. Consistent with this, brightfield morphometric parameters: aggregate number, area, and aspect ratio have been shown to correlate with bacterial growth rate, confirming that imaging data carry substantial biological signal beyond qualitative validation (Arbués et al. 2025).

Although experimental variability is inevitable due to the biological heterogeneity of donor-derived systems, it may also provide an opportunity rather than simply representing a limitation. Drug efficacy can be assessed across a spectrum of GLS structures and immune conditions, potentially yielding insights into both antimicrobial potency and host-pathogen dynamics. By combining greater complexity with experimental accessibility, GLS systems serve as a promising platform for evaluating drugs targeting not only bacterial replication but also host-mediated processes such as immune modulation and intracellular persistence.

### Effect of GLS size on drug efficacy

Reliable comparison of results across experimental setups is essential in drug discovery to generate robust datasets that can inform compound(s) progression. In GLS assays, CFU counts are generally considered the primary readout, while aggregate size is often treated merely as a qualitative indicator for assay validity. However, GLS size distribution can vary significantly between experiments, raising two critical questions:

1. Does GLS size influence drug activity on the bacterial population?
2. Does the bacterial burden within each GLS depend on its size?

If the answer to one or either question is affirmative, then GLS size distribution becomes more than a qualitative indicator, it becomes a determinant of experimental reproducibility. In such a scenario, two experiments should be considered biologically equivalent for measuring drug clearance if they exhibit not only similar CFU counts but also comparable GLS size distributions. Neglecting to account for size variability could introduce significant confounding effects that limit the reliability and comparability of experimental outcomes. Moreover, systematic analysis of size-dependent effects could provide valuable insights into the structural and functional characteristics of GLS themselves.

While systematic measurements are currently lacking, and only isolated observations are available (Silva-Miranda et al. 2015), both questions can nevertheless be explored through a minimal theoretical framework. From a biological standpoint, the second question appears more straightforward. In most GLS assays, the experimental protocol ensures adequate monocyte infection. Since GLS form and persist until their eventual breakdown, it is reasonable to assume that the number of bacteria contained within the GLS correlate with the number of macrophages they comprise. Indeed, being both abundant and voluminous, macrophages largely determine GLS size and, consequently, the maximal bacterial load. The first question is more complex. In vitro GLS differ structurally from *in vivo* granulomas since they do not form compact structures or necrotic cores (Kapoor et al. 2013, Elkington et al. 2019), which are features known to impede tissue penetration of drugs (Dartois 2014, Prideaux et al. 2015, Karakitsios et al. 2025). Consequently, drugs are expected to diffuse through GLS more effectively than through their *in vivo* counterparts. Regardless, penetration alone is not sufficient to generate size-dependent effects. Drug action may also vary due to cellular differentiation states within GLS, as compared to isolated macrophage cultures. For instance, the appearance of foamy macrophages in *in vitro* systems (Peyron et al. 2008) has been associated with dormant bacterial phenotypes and induced rifampin tolerance (Kapoor et al. 2013). This reasoning suggests that GLS size may influence drug efficacy against *Mtb* through both physical (variations in drug penetration) and biological (diverse cellular states) mechanisms.

To examine the implications of this potential size dependency, theoretical conditions should be established under which drug efficacy would remain size independent. This scenario would require:

- the number of bacteria per GLS to be independent of size;
- intra-GLS drug concentration to be independent of its size;
- uniform bactericidal or bacteriostatic effects across macrophage phenotypes, or phenotype distributions invariant with size.

Such conditions are unlikely to hold in practice. Therefore, GLS size is expected to influence experimental outcomes. To illustrate the implications of this dependency, we consider a minimal theoretical model based on four simplifying assumptions: (i) bacterial load scales with GLS volume; (ii) the initial load is identical across experiments; (iii) bacterial growth follows exponential dynamics and; (iv) the net rate of change depends on GLS size (see Supplementary data B for derivation). Within this simplified model, it is possible to clarify the implications of size dependency with a numerical example that compares two experimental configurations: small GLS (A) and large GLS (B). These are identical in protocol and drug exposure and differ only in the number and size of GLSs present in the well (Fig. 5). Experiment A contains two small GLS, each with radius ${{R}_A} = \ 100\ \mu m$, while Experiment B contains one large GLS with radius ${{R}_B}$ chosen so that the initial total bacterial load, $M( {R,\ {{t}_0} = 0} ),$ is preserved by volume scaling, i.e. the relation between the radii is: $2\cdot R_A^3 = \ R_B^3$, from which we can calculate ${{R}_B} = \ {{2}^{\frac{1}{3}}}\cdot{{R}_A} \approx \ 125.99\ \mu $.

**Figure 5:**
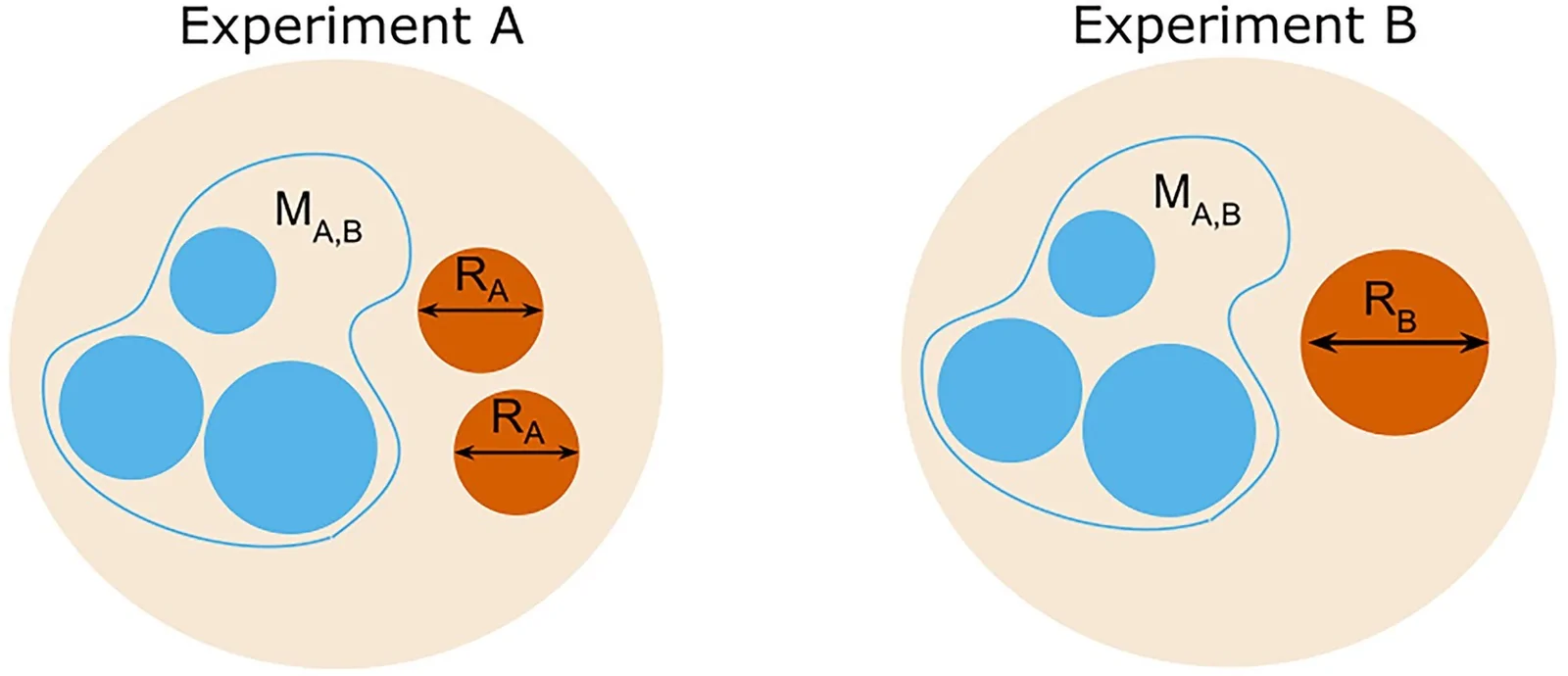
Schematic illustration of two GLS experiments with different size configurations but identical total initial bacterial load. Both experiments contain the same set of GLS, shown in blue, and differ only in the aggregates shown in orange: Experiment A contains two small GLS of radius RA, and Experiment B a single large GLS of radius RB. The radii are related by RB = RA·2^(1/3), so that the two small aggregates of Experiment A occupy the same total volume as the single large aggregate of Experiment B. Because the bacterial load is assumed to scale with volume, the two experiments start from the same total number of bacteria and differ only in how those bacteria are distributed in space.

The number of bacteria in a GLS scales with its volume, *M* (*R*, 0) = *κ R*^3^. The initial load can be calculated, $M( {{{R}_A},0} )\ = \ M( {{{R}_B},0} )\ = \ 2.00 \times {{10}^6}\ CFU$ by assuming κ = 1 CFU/µm³, without loss of generality. Under drug exposure, the bacterial number M(R, t) follows exponential dynamics governed by the net rate $v( R )\ = \ r\ - \ k( R )$:


\begin{eqnarray*}
M\left( {R,t} \right)\ = \ M\left( {R,0} \right)\cdot{{e}^{v\left( R \right)\cdot t}}.
\end{eqnarray*}

For simplicity, we consider a fixed *r* that does not depend on the radius so that the only dependence of the size of the GLS is in the killing parameter *k*. We set the exposure time to $t\ = \ 6$ days and the replication rate $r\ = \ 0.30\ da{{y}^{ - 1}}$. To represent the diminished drug penetration and reduced killing capacity in larger aggregates, we set ${{k}_A} > \ {{k}_B}$ as reported in Table 4. To quantify the bactericidal capacity derived from these dynamics, we define a simple indicator of bacterial killing over the observation window, the Kill Fraction (KF):


\begin{eqnarray*}
KF\ = \ \frac{{\ M\left( {R,\ {{t}_0}} \right) - \ {\mathrm{M}}\left( {{\mathrm{R}},{\mathrm{\ }}{{{\mathrm{t}}}_{\mathrm{f}}}} \right)}}{{{\mathrm{M}}\left( {{\mathrm{R}},{\mathrm{\ }}{{{\mathrm{t}}}_0}} \right)}}\ = \ 1\ - \ {{S}_f},
\end{eqnarray*}

where $M( {R,\ {{t}_0}} )$ and $M( {R,\ {{t}_f}} )$ are the total bacterial counts at the beginning and end of the experiment, and ${{S}_f} = \frac{{M( {R,\ \ {{t}_f}} )}}{{M( {R,\ {{t}_0}} )}} = \ \frac{{{{M}_f}}}{{{{M}_0}}}$ is the residual fraction at $t\ = \ 6$ days. Higher KF values correspond to stronger overall killing. After substituting the parameters into the model, the predicted residual fractions after six days are ${{S}_{f,\ A}} = {{e}^{ - 0.15*6}} \approx \ 0.407$ and ${{S}_{f,\ B}} = {{{\mathrm{e}}}^{ - 0.05{\mathrm{*}}6}} \approx \ 0.741$. These values indicate that, under identical initial load and drug exposure, bacterial survival is substantially higher in the configuration containing larger GLS. Correspondingly, the total bacterial counts at day 6 are ${{M}_{f,A}} = \ 8.12 \times {{10}^5}$ and ${{M}_{f,B}} = \ 1.48 \times {{10}^6}$. Using these values, the kill fraction is $K{{F}_A} \approx \ 0.593$ for experiment A and $K{{F}_B} \approx \ 0.259$ for experiment B. Despite identical initial loads and identical drug protocols, the configuration with large GLS yields a significantly lower KF. Specifically, the bactericidal capacity is more than two-fold higher in the small GLS configuration ($\frac{{K{{F}_A}}}{{K{{F}_B}}} \approx \ 2.3$). Thus, structural differences alone can lead to drastically different quantitative outcomes (Fig. 6).

**Figure 6:**
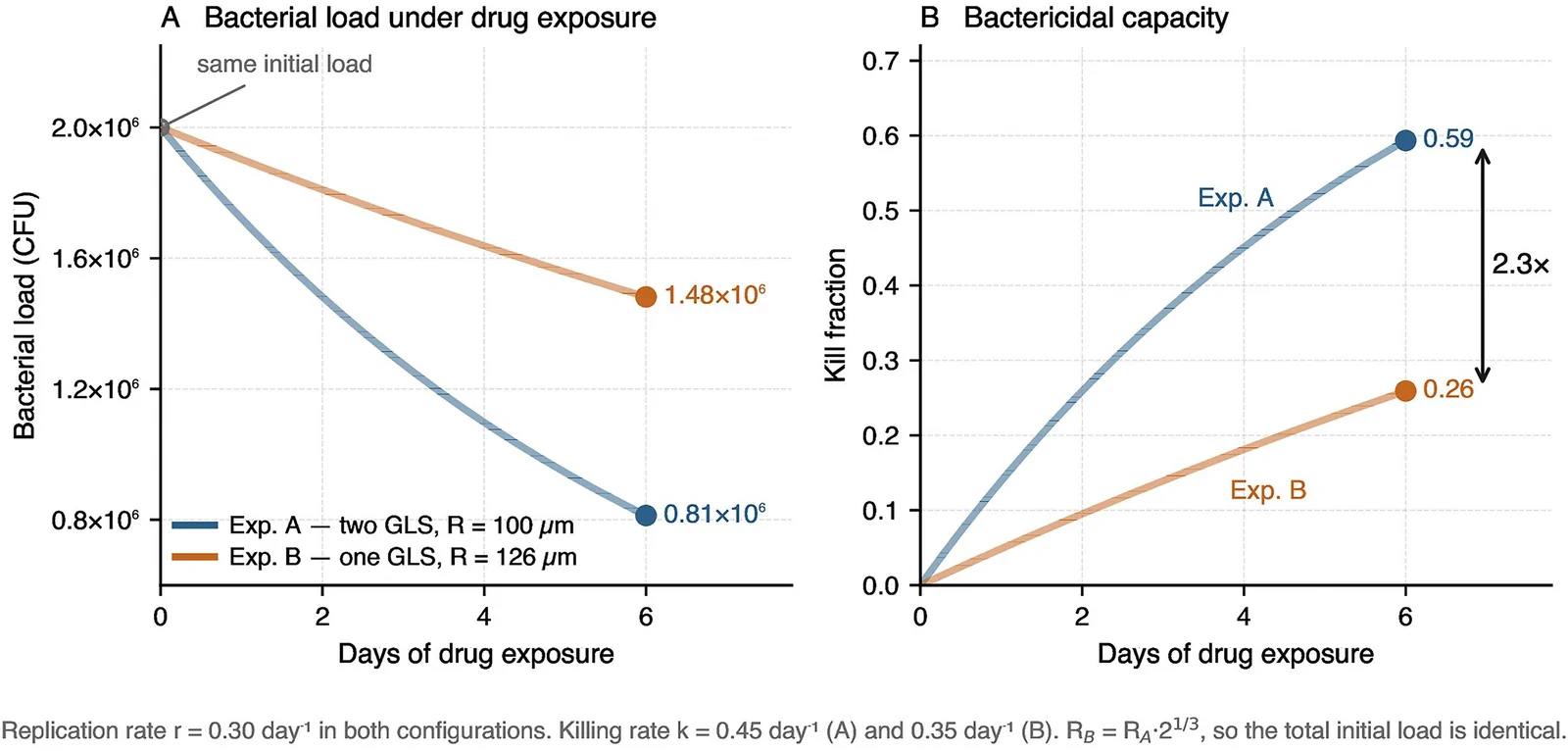
Predicted drug effect in the two theoretical configurations of Fig. 5. (A) Total bacterial load, in colony-forming units (CFU), during six days of drug exposure. Both configurations start from the same load of 2 × 10⁶ CFU. (B) Kill fraction, defined as the proportion of the initial bacterial population removed by the drug. Under identical drug exposure, the configuration made of small GLS reaches a kill fraction 2.3 times higher than the configuration containing a single large one. Model parameters (Table 4): replication rate 0.30 day⁻¹ in both configurations; killing rate 0.45 day⁻¹ in Experiment A and 0.35 day⁻¹ in Experiment B.

**Table 4:** Parameter values used for the theoretical Experiments A and B (reaction–diffusion model, Section 6).

Configuration	Radius	Killing rate (k)	Net rate (v = r − k)
**Exp. A**	RA = 100 µm	kA = 0.45 day⁻¹	vA = −0.15 day⁻¹
**Exp. B**	RB ≈ 126 µm	kB = 0.35 day⁻¹	vB = −0.05 day⁻¹

*r, growth rate; k, killing rate; v, net rate (v = r − k)*.

It should be emphasized that these Kill Fraction values are not experimental measurements but outputs of a minimal, deterministic *in silico* model based on deliberately simplified assumptions. Their purpose is illustrative: to express in quantitative terms a size dependence that is biologically plausible and to show how neglecting it could bias efficacy estimates. Systematically recording GLS size alongside CFU counts would allow the magnitude of this effect to be quantified *in vitro*.

In this context, it is possible to derive a more general framework to calculate the bactericidal effect of the drug, defined as:


\begin{eqnarray*}
E = \frac{{N\left( {{{t}_f}} \right)}}{{N\left( {{{t}_0}} \right)}}.
\end{eqnarray*}

Here, *N* denotes the sum of bacterial loads across all individual granulomas (*M*) within the well. Since the experiments are designed to have identical initial counts, ${{N}_{A}}( {{{t}_{0}}} ) = {{N}_B}( {{{t}_{0}}} ),$ any difference between their predicted efficacies ${{E}_A}$​ and ${{E}_B}$​ must result from a divergence in their final counts. It is straightforward to show mathematically that the difference, *Δ E( c )*, can be expressed as:


\begin{eqnarray*}
{\mathrm{\Delta E}}\left( c \right) = \left[ {\frac{{2M\left( {{{R}_A}} \right)}}{{{{N}_0}}}} \right]*\left\{ {\exp \left[ {{{v}_A}\left( {{{t}_f} - {{t}_0}} \right)} \right] - \exp \left[ {{{v}_B}\left( {{{t}_f} - {{t}_0}} \right)} \right]} \right\}.
\end{eqnarray*}

If the net killing rate *v( R )* is size-dependent, then the rate in the smaller GLS of Experiment A, ${{v}_A}$​, will differ from that of the larger ones in Experiment B, ${{v}_B}$​. As in the example, final bacterial counts will diverge, meaning that ${{N}_{A}}( {{{t}_{f}}} )\ \not\ne {{N}_B}( {{{t}_{f}}} )$, and predicted efficacies will also be different: ${{E}_A}\ \not\ne\ {{E}_B}$.

In practical experiments, the killing rate *v* is not known *a priori*, although it is the very parameter to be estimated as an indicator of the intrinsic effect of the drug. The reasoning presented here suggests that GLS size distribution may act not merely as another variable, but as a structural determinant that could bias this estimation. An observed difference in efficacy might be incorrectly attributed to the clearance capacity of the compound, when in fact it originates from the physical configuration of the system. For this reason, recording the size distribution associated with each CFU count appears to be critically important. Although such measurements may be challenging to perform in practice, they provide a comprehensive picture of the underlying infection dynamics and offer valuable insights into the interaction between the drug and newly formed granulomas.

Although this deterministic exercise does not address measurement uncertainty, it highlights critical experimental issues that variability might otherwise obscure, and that must be considered when designing, interpreting, and comparing GLS experiments. The full derivation, together with a more general formulation incorporating continuous size distributions and size-dependent drug penetration, is provided in Supplementary data B, offering a conceptual foundation for more comprehensive modeling of GLS-based assays.

### *In silico* modeling of GLS

Numerous mathematical and computational models have been developed to study the formation and evolution of granulomas *in vivo*, yet relatively little attention has been directed towards modeling *in vitro* GLS. Existing models are often based on agent-based frameworks, differential equations, or hybrid approaches and focus primarily on tissue-scale immune responses, including cell recruitment, cytokine dynamics, and the emergence of necrotic cores (Segovia-Juarez et al. 2004, Cilfone et al. 2013, Gideon et al. 2015, Fors et al. 2020). These models aim to replicate the complexity of host–pathogen interactions within organs such as the lung or lymph nodes, frequently relying on animal data or broad immunological assumptions. By contrast, *in vitro* GLS experiments offer a fundamentally different modeling opportunity. These systems are experimentally tractable, tightly controlled, and quantitatively accessible. Parameters such as initial MOI, cell composition, cytokine levels, and CFU outputs can be measured and systematically perturbed. Nevertheless, computational approaches specifically tailored to these *in vitro* systems remain scarce.

*In silico* modeling of GLS formation and behavior could address several open questions. Such models could elucidate the microscopic mechanisms underlying immune cell aggregation and structure stabilization. They could further evaluate how variables including donor variability, infection dose, or well geometry impact the resulting GLS distribution, size, and bacterial burden. Even relatively simple formulations—such as agent-based or cellular automaton models—would be informative when calibrated against experimental outputs, including CFU trajectories, aggregate size distributions, and cytokine profiles over time (Read et al. 2012, Bowness et al. 2018).

Computational models could also support the identification of optimal experimental conditions, such as MOI ranges conducive to stable GLS formation, or temporal windows for drug administration and measurement to maximize interpretability. *In silico* simulations can additionally serve as controlled platforms for quantifying biological variability and testing hypothetical interventions prior to laboratory implementation, thereby improving experimental design and reducing redundant experimentation (Cilfone et al. 2013).

Finally, the integration of *in silico* simulations with experimental GLS platforms could enable iterative refinement: models informed by empirical data can generate predictions, which in turn can be tested experimentally and used to refine model assumptions. This approach holds promise not only to improve reproducibility and deepen mechanistic insight, but also to enable virtual screening of treatment conditions, a significant aspect when evaluating drug combinations due to the relatively limited experimental throughput capacity of the GLS assay. In doing so, it contributes to bridging the gap between simplified *in vitro* models and complex *in vivo* systems, ultimately reinforcing their role within drug discovery pipelines.

## Conclusions

This review examined the biological relevance, quantitative behaviour and translational potential of PBMC-derived GLS, including their predictive value for anti-TB drug development. GLS reproduce a defined subset of granuloma-associated processes rather than the full complexity of granuloma biology. Infection triggers the differentiation of monocytes into macrophages, while lymphocytes provide the cues that organize them into three-dimensional aggregates, with a macrophage-rich core and lymphocytes at the periphery. The emerging structures include differentiated cell types such as epithelioid cells, multinucleated giant cells and foamy macrophages. Moreover, the response follows an organized sequence, with an early pro-inflammatory phase (TNF-α, IFN-γ, IL-6, IL-12) followed by an IL-10–associated regulatory shift, and the levels of TNF-α and IFN-γ have been reported to correlate with the number and size of the resulting structures.

As a quantitative assay, GLS behave more consistently than the diversity of published protocols would suggest. Despite the different experimental settings, designed to address different questions, bacterial growth follows a conserved trajectory, with an approximately 2-log increase, even though the multiplicity of infection varies widely and aggregate diameters typically range between 100 and 200 µm. Comparison across studies is often limited by the specific quantities that are measured and the metrics used. CFUs are commonly quantified, but some studies use luminescence or GFP imaging, with drug testing studies which may report only MIC values. Moreover, measurements are often reported only at the end of the experiment rather than as time courses. Aggregate number and morphology are seldom quantified, and cytokine data are available for only a few studies. Including a small, shared set of descriptors—such as bacterial load over time and the distribution of aggregate sizes within the same culture format—would enable cross-study comparisons and meta-analyses. Most of these quantities are already generated during the course of the experiments.

Aggregate size deserves particular attention among these descriptors. The three-dimensional microenvironment of these structures induces dormant, drug-tolerant bacterial phenotypes (Kapoor et al. 2013) and, as well documented *in vivo* (Dartois 2014, Prideaux et al. 2015), may impose physical barriers through restricted diffusion. These factors lead to bidirectional shifts in measured drug activity compared to monolayer systems: compounds may lose potency due to access constraints, while agents like pyrazinamide and isoniazid, which target stressed or non-replicating bacilli, exhibit enhanced activity (Silva-Miranda et al. 2015, Bielecka et al. 2017). As shown by a minimal theoretical model, structural configuration alone can result in a greater than two-fold difference in kill fraction between systems with identical bacterial loads. Failure to record geometric parameters may lead to the misattribution of structural effects to a compound’s intrinsic potency. While GLS offer a cellular context better suited to the evaluation of host-directed therapies, they remain underutilized in favor of standardized but oversimplified models, such as THP-1 monolayers, which fail to replicate these structural and phenotypic complexities (Bielecka et al. 2017, Palucci and Delogu 2018, Elkington et al. 2019).

### Limitations for drug and host-directed-therapy evaluation

The advantages outlined are bounded by limitations of two kinds. The first are structural. GLS develop no necrotic or caseous core and no mature tissue architecture, and the adaptive arm of the response is largely missing: neutrophils are lost during PBMC isolation, and antigen-experienced T and B cells are present only when the donor has been previously exposed to *Mtb*. The model is therefore uninformative for regimens intended to sterilize established, necrotic lesions, and its long cultures, low throughput and biosafety level 3 requirements limit the scale at which it can be applied. The second are a matter of what has not yet been done. No validated *in vitro*–*in vivo* correlation links GLS readouts to granuloma behaviour in animals or patients, so the model is not predictive of clinical efficacy as things stand; measured potency depends on the host cell, the culture format, the timing, the donor, and the size of the aggregates, variables that are not intrinsic flaws but are not yet systematically recorded or controlled; and direct drug-efficacy evidence remains confined to few exploratory studies (Palucci et al. 2020, 2023, Bianco et al. 2023), a reflection of how little the model has been applied rather than of what it can measure.

Taken together, GLS are best understood not as a general-purpose efficacy assay but as a model fit for particular questions: dissecting the early host–*Mtb* encounter, which is difficult to access *in vivo*; evaluating host-directed and immune-modulating therapies; examining how the immune microenvironment drives drug tolerance; and resolving strain- and donor-dependent determinants of outcome. Realizing this potential will depend on standardized reporting, on multi-laboratory benchmarking with reference anti-TB drugs (isoniazid, rifampin, pyrazinamide) and direct comparison with simpler assays, on integration with computational modelling, and on establishing the *in vitro*–*in vivo* correlation the field still lacks (Lin et al. 2006, Dorhoi and Kaufmann 2014)—the steps that would let GLS occupy, with confidence, the space between oversimplified monocultures and complex animal models.

## Supplementary Material

fuag053_Supplemental_Files
