# Supplementary material for: Granuloma-like structures in tuberculosis a critical review of their biology, quantitative behavior, and translational use
Source: FEMS Microbiol Rev. 2026 Oct 21;50:fuag053. doi: 10.1093/femsre/fuag053 (PMC13741237; doi:10.1093/femsre/fuag053)
Supplement: Supplementary file 1 [file fuag053_supplemental_files.zip › Supplementary-data-A.docx]

**Supplementary Data A**

**Supplementary Tables**

This supplementary data compiles the key data tables referenced in Section 6, including cell numbers and proportions (Table S1), bacterial load (Table S2), aggregate sizes and number (Tables S3 and S5), and cytokine profiles (Table S4). Experiment identifiers (E1-E12) are the same used in Table 3 and in Figure 4.

**Experiment key. Data are compiled from the following studies.**

**E1:** Puissegur et al. 2004 — *M. bovis* BCG; 10⁶ PBMC/well; MOI 1:10.

**E2:** Birkness et al. 2007 — H37Rv; 2×10⁵ PBMC + 2×10⁵ autologous macrophages; A: inoculum 10¹ (MOI 1:20,000), B: inoculum 10⁴–10⁵ (MOI 1:20–1:2).

**E3:** Huang et al. 2015 — H37Rv; 10⁶ PBMC/well; MOI 1:100.

**E4:** Arbués et al. 2021 — H37Rv; 2.5×10⁶ PBMC/ml; MOI 1:200; A: 2D, B: 3D (ECM).

**E5:** Seitzer & Gerdes 2003 — 6×10⁵ PBMC/well; A: H37Rv, B: *M. bovis* BCG; MOI 1:150–1:4 (size from uninfected spheroids).

**E6:** Lay et al. 2007 — H37Rv/Erdman/*M. microti*/Beijing; 10⁶ PBMC/well; MOI 1:1000.

**E7:** Guirado et al. 2015 — H37Rv; 2×10⁶ PBMC/ml (2×10⁵ monocytes); MOI 1:1; A: naive donors, B: LTBI donors.

**E8:** Palucci et al. 2020 — PBMC (~10⁵ monocytes); MOI 1:1; A: H37Rv, B: *Mtb* H3.

**E9:** Palucci et al. 2023 — *M. abscessus*; PBMC (~10⁵ monocytes); MAB-S MOI 1:1, MAB-R MOI 1:10.

**E10:** Kapoor et al. 2013 — H37Rv; 5×10⁵ PBMC/well; MOI 1:0.1.

**E11:** Arbués et al. 2025 — 14 clinical strains (lineages L1–L5); 1.25×10⁶ PBMC/well; MOI 1:200.

**E12:** Silva-Miranda et al. 2015 — H37Rv-GFP; 3.5×10⁵ PBMC/well; MOI not reported.

**Notation.** dN, day N post-infection; cytokine values (Supplementary Table S4) are given in pg/ml.

**Supplementary Table S1: Cell numbers and proportions, ref E6**

| **Inoculum** | **Day** | **Cell numbers per granuloma** | **Cell proportions** | **Cell proportions**  **(Cell influx at days 7 and 14)** | **Cell proportions (No cell influx at days 7 and 14)** |
| --- | --- | --- | --- | --- | --- |
| 200 mycobac-  terial surface antigen-coated beads | 7 | 3-5*10^2 | MON/MAC: ca. 12.4  CD4+: ca. 54.3  CD8+: ca. 14.3  Tγδ: ca. 3.8  LB: ca. 4.7 | MON/MAC: ca. 10.9  CD4+: ca. 55.3  CD8+: ca. 14.8  Tγδ: ca. 3.5  LB: ca. 4.7 | MON/MAC: ca. 10.9  CD4+: ca. 55.3  CD8+: ca. 14.8  Tγδ: ca. 3.5  LB: ca. 4.7 |
| **//  //   //** | 14 | 2-3*10^3 | MON/MAC: ca.12.9  CD4+: ca. 62.9  CD8+: ca. 15.7  Tγδ: ca. 4.8  LB: ca. 4.8 | MON/MAC: ca. 9.7  CD4+: ca. 48.7  CD8+: ca. 18.7  Tγδ: ca. 8.6  LB: ca. 5.8 | MON/MAC: ca. 8.6  CD4+: ca. 53  CD8+: ca. 24.2  Tγδ: ca. 13.5  LB: ca. 2.7 |
| **//  //  //** | 21 | 5-6*10^3 | MON/MAC: ca. 9.5  CD4+: ca. 62.9  CD8+: ca. 15.7  Tγδ: ca. 4.7  LB: ca. 4.3 | MON/MAC: ca. 10.5  CD4+: ca. 53.4  CD8+: ca. 19.9  Tγδ: ca. 5.4  LB: ca. 6.6 | MON/MAC: ca. 13.6  CD4+: ca. 56.9  CD8+: ca. 15.6  Tγδ: ca. 5  LB: ca. 13.9 |

**Unactivated PBMCs**

MON/MAC: ca. 20

CD4+: ca. 43.8

CD8+: ca. 10.5

Tγδ: ca. 3.8

LB: ca. 4.8

**Supplementary Table S2: CFU count**

|  | **E3** | **E4A** | **E4B** | **E7A** | **E7B** | **E8A** | **E8B** | **E11** | **E9 MAB-S** | **E9 MAB-R** |
| --- | --- | --- | --- | --- | --- | --- | --- | --- | --- | --- |
| **Day** | **CFU/ml** | **CFU/ml** | **CFU/ml** | **CFU/ml** | **CFU/ml** | **CFU/10^5 cells** | **CFU/10^5 cells** | **CFU/well** | **CFU/10^6 cells** | **CFU/10^6 cells** |
| 0 | 1.00E+03 |  |  |  |  |  |  |  |  |  |
| 1 | 1.875E+03 | 1.87E+04 | 9.86E+03 | 3.5E+03 | 3.5E+03 |  |  | 2E+03 - 7E+03 |  |  |
| 3 | 4.8E+03 |  |  | 8.7E+03 | 5.7E+03 | 1.0E+05 | 1.0E+05 |  |  |  |
| 4 |  | 1.51E+05 | 7.14E+04 | 2.0E+04 | 1.3E+04 |  |  |  |  |  |
| 5 |  |  |  | 2.3E+04 | 1.4E+04 |  |  |  |  |  |
| 6 | 1.5E+04 |  |  |  |  | 2.2E+05 | 3.2E+05 |  |  |  |
| 7 |  |  |  | 2.8E+04 | 1.7E+04 |  |  |  |  |  |
| 8 |  | 8.39E+05 | 6.11E+05 | 3.8E+04 | 2.6E+04 |  |  | 5E+04 - 5.5E+05 |  |  |
| 9 | 3.5E+04 |  |  |  |  |  |  |  |  |  |
| 12 |  |  |  |  |  | 1.4E+06 | 8.9E+05 |  | ~2E+07 | ~2.5E+07 |

**Supplementary Table S3: GLS sizes**

| **Ref** | **Inoculum (bacilli)** | **AM/PBMC** | **MOI**  **(*Mtb*/cell)** | **Strain** | **d1** | **d3** | **d4** | **d5** | **d6** | **d7** | **d8** | **d9** | **d10** |
| --- | --- | --- | --- | --- | --- | --- | --- | --- | --- | --- | --- | --- | --- |
| E2 | 10^1 | 2x10^5 MA  +  2x10^5 non-adherent PBMC | 1:20,000 (D) | H37Rv | 73.5±30 |  |  |  |  |  |  | 179±37 |  |
| E2 | 10^4 –10^5 |  | 1:20 - 1:2 (D) |  | 155±66 |  |  |  |  |  |  | 245±64 |  |
| E3, fig 3 | 10^4 | 10^6 PBMC | 1:100 | H37Rv |  | ~50 |  |  | ~150 |  |  | ~200 |  |
| E1, img.5 | 10^5 | 10^6 PBMC | 1:10 (D) | BCG | ~15 |  |  | ~40 |  |  |  | ~150 |  |
| E5, uninfected | 0 | 6x10^5 PBMC agarose-coated wells | n.a. (uninfected) |  |  |  | 597 ± 45 |  |  | 589 ± 38 |  |  |  |
| E4, fig 1B  2D | 1.25x10^4 (D) | 2.5x10^6 PBMC | 1:200 | H37Rv/a |  |  | ~100 (max) |  |  |  | ~200 (max) |  |  |
| E4, fig 1B  3D |  |  |  |  |  |  | ~20 |  |  |  | ~100 (max) |  |  |
| E11, area in um^2 -> diameter | - | 1.25x10^6 PBMC | 1:200 | 14 strains (L1-L5) |  |  |  |  |  | 66 - 100 |  |  |  |
| E8A | - | ~1×10⁵ monocytes | 1:1 | H37Rv |  |  |  |  |  |  |  |  | ~126 |
| E8B | - | ~1×10⁵ monocytes | 1:1 | *Mtb* H3 |  |  |  |  |  |  |  |  | ~107 |
| E9 | - | ~1×10⁵ monocytes | 1:1 | *M. abscessus* MAB-S |  |  |  |  |  |  |  |  | ~94 |
| E9 | - | ~1×10⁵ monocytes | 1:10 | *M. abscessus* MAB-R |  |  |  |  |  |  |  |  | ~93 |

**Supplementary Table S4: Cytokines**

|  | **E7** | | | | | | **E3** | | **E2** | | | | **E9 MAB-R** | **E9 MAB-S** |
| --- | --- | --- | --- | --- | --- | --- | --- | --- | --- | --- | --- | --- | --- | --- |
| **Days** | **IFN-γ** | **TNF-α** | **IL-2** | **IL-10** | **IL-12** | **IL-13** | **TNF-α** | **IL-10** | **IFN-γ** | **TNF-α** | **IL-6** | **IL-8** | **IL-1b / IL-6 / TNF-a / IL-8** | **IL-1b / IL-6 / TNF-a / IL-8** |
| 1 | 16 (139) | 465 (658) | n.d. (56) | 67 (110) | 10 (90) |  |  |  | 404  ± 0 | 6131  ± 5420 | 12752  ± 7963 | 103553  ± 13266 |  |  |
| 2 |  |  |  |  |  |  |  |  | 5512  ± 6724 | 8414  ± 7807 | 31479  ± 20191 | 259962  ± 158128 |  |  |
| 3 | 108 (437) | 258 (465) | n.d. (37) | 52 (60) | 161 (664) | n.d. (31) | 12000 | 700 |  |  |  |  |  |  |
| 4 | 150 (493) | 168 (735) |  | 26 (88) | 151 (815) | n.d. (169) |  |  |  |  |  |  |  |  |
| 5 | 232 (514) | 581 (955) |  | 48 (93) | 211 (815) | n.d. (71) |  |  | 31810  ± 39284 | 8745  ± 4835 | 56841  ± 16675 | 313253  ± 126042 |  |  |
| 6 | 78 (452) | 542 (1174) |  | 64 (75) | 140 (875) | n.d. (404) | 30000 | 1400 |  |  |  |  |  |  |
| 7 | 93 (555) | 310 (774) |  | 36 (56) | 110 (674) | n.d. (35) |  |  |  |  |  |  |  |  |
| 8 | 175 (463) | 103 (1316) |  |  | 60 (181) |  |  |  |  |  |  |  |  |  |
| 9 |  |  |  |  |  |  | 24000 | 3000 | 25429  ± 31528 | 5925  ± 5130 | 66843  ± 34499 | 424745  ± 232197 |  |  |
| 12 | 268 (534) |  |  | 17 (17) | 252 (926) | n.d. (35) |  |  |  |  |  |  | 32,132 / 14,862 / 49,896 / 115,314 | 57.5 / 188 / 1,658 / 63,680 |

**Supplementary Table S5: GLS number**

| **Ref** | **Add info** | **d3** | **d4** | **d6** | **d7** | **d8** | **d9** | **d10** |
| --- | --- | --- | --- | --- | --- | --- | --- | --- |
| E3 (from text), fig.5B  AVG 20 random,  Image size ~ 1100x800  LPSx100 | control | 4.1 |  | 5.05 |  |  |  |  |
|  | IFN-g + LPF pre treated | 9.15 |  | 10.95 |  |  |  |  |
|  | IL-4 pre treated | 3.20 |  | 3.75 |  |  |  |  |
| E5 |  |  | 1.2 ± 0.6 |  |  |  |  |  |
| E4, fig 1b  2D  Image size: ~700x700,  Zoom: 10X |  |  | 7-10 |  |  | 10-15 |  |  |
| E4, fig 1b  3D  Image size: ~700x700,  Zoom: 10X |  |  | Many small |  |  | ~8 |  |  |
| E8A, fig 6 Magnification ×40 |  |  |  |  |  |  |  | 8/field |
| E8A, fig 6 Magnification ×40 | Cystamine 400 μM at day 6 p.i. |  |  |  |  |  |  | 4/field |
| E8A, fig 6 Magnification ×40 | Cysteamine 800 μM at day 6 p.i. |  |  |  |  |  |  | 4/field |
| E8A, fig 6 Magnification ×40 | INH MIC at day 6 p.i. |  |  |  |  |  |  | 2/field |
| E9, fig 3 | *M. abscessus* S |  |  |  |  |  |  | 30 |
| E9, fig 3 | *M. abscessus* R |  |  |  |  |  |  | 35 |
| E8B, fig 6F | *Mtb* H3 (clinical strain) |  |  |  |  |  |  | 5/field |
| E11, fig 3 | 14 clinical strains, median |  |  |  | 22/field |  |  |  |
