# Supplementary material for: Granuloma-like structures in tuberculosis a critical review of their biology, quantitative behavior, and translational use
Source: FEMS Microbiol Rev. 2026 Oct 21;50:fuag053. doi: 10.1093/femsre/fuag053 (PMC13741237; doi:10.1093/femsre/fuag053)
Supplement: Supplementary file 1 [file fuag053_supplemental_files.zip › Supplementary-data-B.docx]

**Supplementary Data B**

**On the Generalization and Theoretical Soundness of the GLS Size–Efficacy Model**

Two experimental wells can be considered identical in experimental protocol and drug exposure but differing in aggregate structure, i.e., well A contains two small GLS of radius $R_{A}$, while well B contains one large GLS of radius $R_{B}$ (Figure 7). The model is then built on four key hypotheses (Table 7):

1. Size–Bacteria Scaling: reflecting the biological expectation that larger GLS would host more bacteria, the number of *Mtb* cells in a granuloma, , is assumed to be proportional to its volume, or .$M\left( R \right)M\left( R \right)\propto R^{3}$
2. Reproducibility Constraint: to ensure comparability between experiments, the initial total bacterial load, $N_{0}$, is set to be identical in both wells. In this scenario, this requires bacterial load of the two small GLS in well A to be equal to the single large one in well B: $2M\left( R_{A} \right)=M\left( R_{B} \right)$. Since bacterial load is assumed to scale with volume (which is proportional to $R^{3}$), this condition implies a direct relationship between the two radii: $2*R_{A}^{3}=R_{B}^{3}$.
3. Exponential Dynamics: bacterial load within a granuloma is assumed to evolve according to exponential dynamics, described as $M\left( t_{f} \right)=M\left( R \right)*e^{\left\{ v\left( R \right)\left( t_{f}-t_{0} \right) \right\}}$.
4. Size-Dependent Net Rate: the parameter $v\left( R \right)$ depends on R and represents net rate of bacterial change i.e., $r(R)-\kappa(R)$, where r and κ are respectively the replication and the killing rate. This dependency captures the combined biological possibilities that both drug penetration and cellular states—hence drug tolerance—vary with GLS size.

***Table 7****: Model Assumptions*

| Assumption | Description |
| --- | --- |
| Size–bacteria scaling | Bacterial count within a GLS scales with its volume, i.e., M(R)∝R3 |
| Post-day 6 stability | GLS sizes are assumed to be fixed from day 6 onward, coinciding with drug administration. |
| No necrosis or core structure | GLSs are modeled as homogeneous, compact structures without necrotic centers, in line with *in vitro* observations. |
| Uniform drug exposure | Drug concentration is assumed to be spatially uniform across the well; variation in efficacy arises from structural or phenotypic differences. |
| No inter-GLS bacterial migration | Bacteria are assumed to remain within their originating GLS; there is no bacterial movement between structures. |
| Equal initial CFU across experiments | All comparative experiments are initialized with the same total bacterial burden. |
| Size-dependent growth rate v(r,c)v(r, c) | The net growth or decay rate may vary with GLS size due to differences in oxygenation, cell state, or drug penetration. |
|  |  |

In both experiments, the total number of *Mtb* cells present in the well is estimated through CFU counts. Since CFUs are obtained by homogenizing and plating the entire well content, they provide a bulk measurement of bacterial load without preserving information about the number or size of individual granulomas. For simplicity, it is assumed that by , GLS sizes stabilize and remain constant. is defined as , the starting point for drug administration, and drug efficacy is evaluated within a short time window, before GLS degradation occurs due to natural cell death. The number of *Mtb* cells within a granuloma, , is assumed to be directly proportional to its size. As well, it is assumed that identical GLS size distributions result with the same total *Mtb* load, although different GLS configurations may still yield equivalent CFU counts. For a granuloma of radius , bacterial content is proportional to its volume: $Nday X Day Xt_{0}MR$

$$M\left( R_{A} \right)= kR_{A}^{3}$$

where is a scaling constant. This reflects the hypothesis that larger GLS can accommodate more macrophages, which serve as the primary sites for *Mtb* replication. Since GLS are typically dominated by macrophages, with minimal necrotic or acellular cores, volume-based scaling represents a plausible first-order approximation. While the precise functional form may vary depending on biological or spatial constraints, the essential assumption is that bacterial load is a monotonic function of GLS size.$k$

Since $R_{A}< R_{B}$, it follows that:

$$M\left( R_{A} \right)< M\left( R_{B} \right)$$

and the total bacterial counts at *t_0_* are:

$$N_{A}\left( t_{0} \right)= M_{\left\{ A,B \right\}}+ 2M\left( R_{A} \right)$$

$$N_{B}\left( t_{0} \right)= M_{\left\{ A,B \right\}}+M\left( R_{B} \right)$$

where $M_{A,B}$ represents the contribution from the common population of GLS in the two experiments. To achieve reproducibility, it is further assumed that:

$$N_{A}\left( t_{0} \right)= N_{B}\left( t_{0} \right)= N_{0}$$

which implies:

$$2M\left( R_{A} \right)= M\left( R_{B} \right)$$

Thus, although GLS size distributions differ, the initial *Mtb* count is identical. After drug administration at time *t_0_*, *Mtb* numbers evolve due to replication and drug-mediated killing. Assuming exponential dynamics, the bacterial load in a granuloma of size at the final time is:$Rt_{f}$

$$M\left( t_{f} \right)= M\left( R \right)*\exp\left[ v\left( t_{f}- t_{0} \right) \right]$$

where $v$ is the net rate of change (growth minus death). For an effective drug, then $v< 0$, and bacterial numbers will decrease over time. Assuming $v$ is known, drug efficacy can be computed as*:*

$$E\left( c \right)=\frac{N\left( t_{f},c_{f} \right)}{N\left( t_{0},c_{0} \right)}$$

The final bacterial loads in each experiment are:

$N_{A\left( t_{f},c_{f} \right)}= M_{\left\{ A,B \right\}\left( t_{f} \right)}+ 2M\left( R_{A},t_{f} \right)= M_{\left\{ A,B \right\}\left( t_{f} \right)}+ 2M\left( R_{A} \right)*\exp\left[ v_{A}\left( t_{f}-t_{0} \right) \right]$

$$N_{B\left( t_{f},c_{f} \right)}= M_{\left\{ A,B \right\}\left( t_{f} \right)}+ M\left( R_{B},t_{f} \right)= M_{\left\{ A,B \right\}\left( t_{f} \right)}+ M\left( R_{B} \right)*\exp\left[ v_{B}\left( t_{f}-t_{0} \right) \right]$$

where *v_A_* and *v_B_* indicate that aggregate rates may differ if considered GLS are different. Corresponding drug efficacies are:

$$E_{A\left( c \right)}=\frac{\left\{ M_{\left\{ A,B \right\}\left( t_{f} \right)}+ 2M\left( R_{A} \right)*\exp\left[ v_{A}\left( t_{f}-t_{0} \right) \right] \right\}}{\left[ M_{\left\{ A,B \right\}}+ 2M\left( R_{A} \right) \right]}$$

$$E_{B\left( c \right)}=\frac{\left\{ M_{\left\{ A,B \right\}\left( t_{f} \right)}+ M\left( R_{B} \right)*\exp\left[ v_{B}\left( t_{f}-t_{0} \right) \right] \right\}}{\left[ M_{\left\{ A,B \right\}}+ M\left( R_{B} \right) \right]}$$

Using $2M\left( R_{A} \right)= M\left( R_{B} \right)$, the difference among measured efficacies becomes:

$$\text{Δ}\text{E}\left( c \right)= \left[ \frac{2M\left( R_{A} \right)}{N_{0}} \right]*\left\{ \exp\left[ v_{A}\left( t_{f}-t_{0} \right) \right]-\exp\left[ v_{B}\left( t_{f}-t_{0} \right) \right] \right\}$$

Thus, if $v$ depends on GLS size ($v=v\left( R \right)$ and $v_{A}\neq v_{B}$), then $\Delta E\left( c \right)\neq0$, meaning efficacy differs between Experiments A and B, despite identical conditions. This difference arises solely from the GLS structure. Conversely, if $v_{A}=v_{B}$, then $\Delta E(c)=0$, and both experiments will yield the same efficacy.

In practice, $v$ is not known *a priori*, as it is usually the parameter to be estimated. The above reasoning shows that such estimates may be biased unless relevant factors such as drug concentration within GLS, GLS maturation and size distribution are independently measured. Incorporating size distribution to CFU counts appears therefore critical to comprehensively picture the underlying dynamics of GLS biology and interpret drug responses.

This simplified model can be generalized into a biologically informed mathematical structure. Rather than assuming a discrete configuration of GLS sizes, a more general approach can be described by a continuous distribution of radii, expressed as $f\left( R \right)$ and normalized such that:

$$\int_{0}^{\infty} f\left( R \right)dR= 1$$

Each granuloma of radius $R$ is assumed to host a number of bacteria proportional to its volume, so that:

$$M\left( R, t \right)= kR_{A}^{3}\cdot exp\left[ v\left( R, c \right)\left( t- t_{0} \right) \right]$$

where is a constant, is the time of drug administration, and is the net growth rate of *Mtb* under drug concentration .$kt_{0} v\left( R, c \right)c$

The growth rate includes both intrinsic bacterial replication and drug-induced killing:

$$v\left( R, c \right)= \lambda\left( R \right)- \kappa\left( R, c \right)$$

where $\lambda\left( R \right)$ depends on factors such as oxygen or nutrient availability, and $\kappa\left( R, c \right)$ reflects drug bactericidal or bacteriostatic effect. Both variables may vary with GLS size due to differential drug penetration, nutrient gradient or cellular composition.

The total bacterial number in the well at time $t$ is then:

$$N\left( t, c \right)= N_{0}\int_{0}^{\infty} \left[ R^{3}/\left\langle R^{3} \right\rangle\right]\cdot exp\left[ v\left( R,c \right)\left( t- t_{0} \right) \right]f\left( R \right)dR$$

where is the initial total *Mtb* load, and ensures that size distribution is properly normalized to account for bacterial contribution.$N_{0}\left\langle R^{3} \right\rangle= \int R^{3}f\left( R \right)dR$

Drug efficacy at time $t$ can then be expressed as:

$$E\left( c \right)=N(t, c)/N_{0}$$

This formulation allows nonlinear dependencies of $v(R, c)$ on size and drug concentration. For instance, a linear size-dependence on drug efficacy can be modeled as:

$$v\left( R,c \right)=v_{0}\left( c \right)- \beta R$$

where accounts for diminished efficacy in large GLS, consistent with structural barriers, foamy macrophage presence, or altered drug availability (Peyron et al., 2008; Kapoor et al., 2013). Substituting linear size-dependency into the integral highlights how differences in —GLS size distribution—can result in measurable differences in the observed efficacy, even under constant experimental conditions. This insight reinforces the central message advanced in the review: quantitative assessment of GLS size distribution is not just desirable, but potentially essential for the correct interpretation of drug efficacy *in vitro*. Moreover, it highlights that commonly used bulk metrics such as CFU counts may conceal important structural differences that influence treatment outcomes.$\beta f(R)$
